# Hemagglutinin Protease HapA Associated With *Vibrio cholerae* Outer Membrane Vesicles (OMVs) Disrupts Tight and Adherens Junctions

**DOI:** 10.1002/jev2.70092

**Published:** 2025-05-25

**Authors:** Palwasha Baryalai, David Irenaeus, Eric Toh, Madeleine Ramstedt, Bernt Eric Uhlin, Aftab Nadeem, Sun Nyunt Wai

**Affiliations:** ^1^ Department of Molecular Biology and Umeå Centre for Microbial Research (UCMR) Umeå University Umeå Sweden; ^2^ The Laboratory for Molecular Infection Medicine Sweden (MIMS) Umeå University Umeå Sweden; ^3^ Department of Chemistry and Umeå Centre for Microbial Research (UCMR) Umeå University Umeå Sweden

**Keywords:** adherens junctions, cholera, outer membrane vesicles, protease, tight junctions, virulence

## Abstract

This study explores the virulence mechanisms of *Vibrio cholerae*, with a particular emphasis on HapA, a zinc metalloprotease delivered via outer membrane vesicles (OMVs). The findings reveal that OMV‐associated HapA disrupts the integrity of tight and adherens junctions in intestinal epithelial cell models more effectively than its purified counterpart, suggesting that association with OMVs substantially potentiates the pathogenic effects of HapA. The study further details the uptake of *V. cholerae* OMVs by epithelial cells, as well as their targeted degradation of key junctional proteins, including claudin, ZO‐1, and β‐catenin. These results highlight the critical role of OMV‐associated HapA in compromising epithelial barrier function. Additionally, the use of spheroids and intestinal organoids in our experiments provides deeper insight into bacterial pathogenesis, offering valuable information for the development of targeted therapeutic strategies.

## 1 Introduction

*Vibrio cholerae*, a Gram‐negative bacterium, is the causative agent of cholera, a severe gastrointestinal disease that poses a significant public health challenge, particularly in developing countries (Weil et al. 2019). Among the over 200 identified serogroups of *V. cholerae*, serogroups O1 and O139 are known for their ability to produce two primary virulence factors: cholera toxin (CT) and the toxin co‐regulated pilus (TCP). These factors are crucial for intestinal colonisation and the progression of cholera (Chatterjee et al. 2009; Konig et al. 2016; Trucksis et al. 1993). In addition to CT and TCP, the O1 and O139 serogroups also produce accessory toxins such as zonula occludens toxin (Zot), vibrio cytolysin (VCC), repeats in toxin (MARTX), accessory cholera enterotoxin (Ace), and cholix toxin (ChxA), enhancing *V. cholerae*'s virulence profile (Chatterjee et al. 2009; Kathuria and Chattopadhyay 2018; Konig et al. 2016; Trucksis et al. 1993). Despite lacking CT and TCP, non‐O1/non‐O139 serogroups can still cause diseases such as skin infections and mild diarrhoea, largely due to these accessory toxins. Moreover, *V. cholerae* produces cholera lectin, a metalloprotease with hemagglutinating and proteolytic activities, identified in strain CA401 and affecting significant targets such as lactoferrin and the A subunit of *Escherichia coli*'s heat‐labile toxin (LT), further contributing to the bacterium's pathogenic capabilities (Finkelstein et al. 1983; Finkelstein and Hanne 1982).

Bacterial proteases, functioning as virulence factors, play a crucial role in facilitating bacterial pathogenesis by causing extensive tissue damage that aids in bacterial entry into host cells. Among these, the hemagglutinin (HA)/protease, HapA, a zinc‐dependent metalloprotease secreted by *V. cholerae*, exhibits a wide range of pathogenic activities in both cell culture and animal models, including the modification of other toxins, degradation of the protective mucus barrier, and disruption of intestinal tight junctions (Benitez and Silva 2016). The protease's action on vital substrates, including mucin, fibronectin and lactoferrin, highlights its critical role in mediating tissue destruction, enhancing host cell penetration and eliciting specific physiological effects, such as inducing haemorrhagic responses and causing changes in target cell morphology (Benitez and Silva 2016; Booth et al. 1983; Miyoshi 2013). HapA's multifaceted role extends beyond its enzymatic functions, acting as an important component in the ‘mucinase complex’ alongside sialidase. This complex plays a critical role in degrading the mucosal barrier, thereby exposing GM1 gangliosides for cholera toxin binding and uptake (Crowther et al. 1987; Stewart‐Tull et al. 1986).

The role of HapA in cholera pathogenesis is further underscored by its ability to process and activate several toxins. These include the A subunit of the cholera toxin, *V. cholerae*'s primary virulence factor, and the cytolytic toxin cytolysin/haemolysin A (VCC/HlyA), which contributes to the diarrheal symptoms of cholera disease (Booth et al. 1984; Nagamune et al. 1996). Additionally, HapA supports the initial stages of colonisation by interacting with chitin oligosaccharide deacetylase, GbpA, enhancing bacterial adhesion and microcolony development on intestinal cells. As the infection advances, the degradation of GbpA by HapA assists in the bacteria's detachment from the host, facilitating their return to aquatic habitats (Jude et al. 2009).

The regulation of HapA expression involves an intricate balance of cell density and environmental signals. At low cell densities, virulence genes are activated, but as cell density increases, the quorum‐sensing regulator HapR represses certain virulence factors while promoting HapA expression. This intricate regulatory mechanism ensures the timely production of HapA, allowing *V. cholerae* to adapt to various ecological niches and lifecycle stages. Environmental factors such as anaerobic conditions and the presence of mucin or bile salts further influence HapA expression, highlighting the bacterium's sophisticated response to external stimuli (Jude et al. 2009; Zhu and Mekalanos 2003). Due to its action on multiple targets, HapA's role in disease is complex and may vary depending on the *V. cholerae* strain (Benitez and Silva 2016). Studies in humans using *hapA* mutants have revealed the pathological effects of HapA during infection (Montero et al. 2023).

Tight junctions (TJs) are specialised intercellular connections present in both epithelial and endothelial cells, essential for maintaining cell polarity by segregating the apical and basolateral membrane domains. Additionally, TJs serve as a selective barrier, regulating paracellular transepithelial transport of solutes and water (Guttman and Finlay 2009; Wu et al. 2000). Zonula occludens‐1 (ZO‐1) proteins are located immediately beneath the cell membrane, forming a scaffolding network that anchors additional tight junction proteins (Stevenson et al. 1986). This network is further associated with transmembrane proteins such as occludin, claudins and junctional adhesion molecules (JAM), which, along with other intracellular proteins, contribute to the structural and functional integrity of the tight junction (Furuse et al. 2002). *V. cholerae* HapA affects Mardin‐Darby Canine Kidney I (MDCK‐I) epithelial cells by impairing their barrier function and altering the structure of ZO‐1 and the F‐actin cytoskeleton (Wu et al. 1996), possibly by targeting occludin degradation, which could initiate signalling pathways impacting the spatial organisation of ZO‐1 and the dynamics of the F‐actin cytoskeleton (Wu et al. 2000).

Bacterial membrane vesicles function as a critical secretion system for both Gram‐negative and Gram‐positive bacteria, enabling the transfer of diverse molecules, including lipids, toxins, nucleic acids and small molecules, to both eukaryotic and bacterial cells (Zlatkov et al. 2021). These vesicles play a vital role in different biological processes, such as modulating host immune responses, biofilm formation and genetic material transfer. Importantly, bacterial vesicles deliver virulence factors directly into target cells, bypassing host defences and disrupting normal cell functions, thereby significantly contributing to bacterial pathogenicity and intercellular communication (Nadeem et al. 2020; Zlatkov et al. 2021).

In our previous studies, we demonstrated that HapA is secreted from *V. cholerae* in association with outer membrane vesicles (OMVs). The OMVs‐associated HapA induced an enterotoxic response in the mouse ileum loop assay, highlighting their biological impact (Mondal et al. 2016). Despite these findings, the specific effects of OMV‐associated HapA on cellular and subcellular structures have yet to be thoroughly investigated. Addressing this gap, our current study delves into a detailed examination of OMV‐associated HapA using a variety of models, such as two‐dimensional (2D), three‐dimensional (3D) and organoid systems, aiming to elucidate its intricate cellular and subcellular dynamics. Our findings revealed that HapA, associated with OMVs, is internalised into the intracellular compartments of the cells via caveolin‐mediated endocytosis or macropinocytosis, leading to the intracellular release of HapA. Once inside, HapA cleaves tight‐ and adherens junction proteins such as ZO‐1, F‐Actin, Claudin, E‐cadherin and β‐catenin. This elucidates the sophisticated mechanisms by which HapA compromises the structural integrity of cellular junctions.

In summary, our research investigates the intricate interplay between the bacterial secreted factor HapA and host cell tight‐ and adherens‐junction proteins, shedding light on the mechanisms underlying bacterial pathogenesis and providing valuable insights into potential therapeutic interventions.

## 2 Materials and Methods

### 2.1 Bacterial Strains and Plasmids

The bacterial strains used in this study included the wild‐type *V. cholerae* strain A1552, its isogenic Δ*hapA*, Δ*hapR*, Δ*hapRΔvcc* and Δ*prtV* mutants. The *V. cholerae* strains were cultured overnight in LB broth at 37°C with shaking. The *E. coli* K12 strain MC1061/pBAD18 vector control and *E. coli* MC1061/p*hapA+* were also used. Both strains were grown at 37°C in LB broth, supplemented with 100 µg/mL carbenicillin and 0.1% L‐arabinose. The purpose of adding L‐arabinose was to induce the expression of the genes carried by the plasmid.

### 2.2 Isolation and Analysis of OMVs From *V. cholerae*

OMVs were isolated from bacterial culture supernatants collected after overnight incubation of bacteria at 37°C as described previously (Wai et al. 2003). Briefly, the bacterial cultures grown at 37°C for 16 h were centrifuged at 5000 × *g* for 30 min at 4°C. Then the supernatants were filtered through a 0.2‐µm pore size sterile Minisart High Flow syringe filter (Sartorius Stedim) and ultracentrifuged at 100,000 × *g* for 4 h at 4°C in a 45 Ti rotor (Beckman). The vesicle pellet was resuspended in phosphate‐buffered saline (PBS), and the suspension was used as the crude OMV preparation. The concentration of OMVs in the suspension was determined based on the protein concentration, which was measured using the BCA protein assay (DCTM protein assay, Bio‐Rad).

Nanoparticle tracking analysis (NTA) was performed using a NanoSight NS500 instrument (Malvern Ltd, Worcestershire, UK) equipped with the NTA (version 3.4) analytical software to determine particle size, with samples diluted in PBS to achieve a particle count of 2 × 10⁸ to 2 × 10⁹ particles per millilitre. NTA was used to visualise individual particles in real time. Using a 1 mL syringe, the OMV samples were injected into the instrument, and five videos (1 min each) were recorded for each sample. Particle count and size distribution were analysed using the NTA software (version 3.4). In addition, OMV samples were further characterised by SDS‐PAGE, electron microscopy and immunoblotting, as described below.

### 2.3 Density Gradient Centrifugation and Dialysis

OptiPrep Density Gradient Medium (Sigma–Aldrich/Merck, Cat# D1556; 20%–40% w/v) was prepared by dilution with Milli‐Q (MQ) water and sterile‐filtered through a 0.2 µm filter to eliminate potential contaminants. The gradient was carefully layered into 10 mL centrifuge tubes, starting with the highest concentration (40%) at the bottom and decreasing to 20% at the top. Tubes were allowed to equilibrate overnight at 4°C. The next day, 500 µL of the sample was gently loaded on top of the gradient and centrifuged at 100,000 × *g* for 20 h at 4°C using a Beckman Coulter LE‐70 Ultracentrifuge.

For dialysis, Slide‐A‐Lyzer Dialysis Cassettes (Thermo Fisher Scientific, Cat# 66380) were used. Cassettes were pre‐soaked in PBS for 5–10 min before loading. Samples were introduced using a sterile 1 mL syringe (BD Plastics, REF# 303172), and any trapped air was removed via the same syringe. The cassettes were then placed vertically in PBS to allow efficient diffusion of OptiPrep through the dialysis membrane. Samples were collected the following morning.

### 2.4 Cloning and Purification of HapA

The *hapA* gene (UniProt code: A0A2P0ZHJ3) was amplified by PCR using genomic DNA extracted from the wild‐type *V. cholerae* strain, A1552. The resulting PCR product was digested with (*Nco*I +*Xho*l) and subsequently inserted into the plasmid pET22b. During this process, the original signal peptide of HapA was substituted with the PelB signal sequence, and a C‐terminal His‐tag was incorporated. The construct was expressed in Rosetta (DE3) cells cultured in LB broth supplemented with 50 µg/mL kanamycin and 34 µg/mL chloramphenicol. Protein expression was induced with 0.2 mM isopropyl 1‐thio‐β‐d‐galactopyranoside (IPTG) when the OD600_nm_ reached 0.6, and the culture was grown overnight at 16°C. After harvesting the cells, the periplasmic fraction was isolated. The fraction enriched with periplasmic proteins was then loaded onto a Ni‐sepharose column (Roche). The column was washed with a buffer containing 50 mM sodium phosphate (pH 8.0), 0.5 M NaCl and 5 mM imidazole. The protein was then eluted using a buffer with 0.3 M imidazole. For further purification, the protein was run on a HiLoad 16/600 Superdex 200 prep‐grade column (Cytiva), pre‐equilibrated with 1X PBS (pH 7.4).

### 2.5 Azocasein Assay

A 1% azocasein solution was prepared in 20 mM Tris‐HCl, pH 8.0, using azocasein from Sigma–Aldrich (Cat. #A2765). Supernatants or OMVs from the bacterial strains were added to the 1% azocasein solution and incubated for 3 h at 37°C. The reaction was then stopped by adding an equal volume of 10% trichloroacetic acid (TCA). After a brief vortex, the samples were incubated on ice for 5 min. Precipitated proteins were removed by centrifugation at 13,000 rpm for 15 min. The supernatants were transferred to a 96‐well plate, and absorbance was measured at 440 nm using a Tecan Spark plate reader.

### 2.6 BCA Assay for Protein Estimation

Protein quantification was carried out using the Pierce BCA Protein Assay Kit (Thermo Scientific, #23225). A 2000 µg/mL albumin solution, provided with the kit, was used as the standard. The working solution was prepared by mixing two reagents at a 1:50 dilution ratio. Standards and samples were incubated with the working reagent in a 96‐well plate for 30 min at 37°C. Absorbance was measured at 562 nm using the Tecan Spark Cyto plate reader.

### 2.7 Cryogenic X‐Ray Photoelectron Spectroscopy (Cryo‐XPS)

The surface chemical composition was analysed using cryo‐XPS on a Kratos Axis Ultra DLD electron spectrometer, following the previously established protocol for OMV samples (Shchukarev et al. 2021). Two biological replicates of OMVs were analysed, with each sample consisting of frozen 5–10 µL drops of vesicle suspension in PBS buffer. Data processing was carried out using CasaXPS to apply charge correction to the binding energy scale, followed by the removal of a Shirley background. Spectral components were then determined using a MATLAB script, as previously described (Ramstedt et al. 2011; Ramstedt and Shchukarev 2016).

### 2.8 Cell Culture and 3D Spheroids

Caco‐2 (ATCC) and HCT8 (ATCC) cells were cultured in 6‐well plates (Thermo Fisher Scientific) at a density of 3 × 10^5^ cells per well. The cells were grown as a polarised layer or a monolayer using Roswell Park Memorial Institute (RPMI) 1640 medium (Sigma–Aldrich) supplemented with 10% foetal bovine serum (FBS), 1% penicillin/streptomycin and non‐essential amino acids. The cultures were maintained at a temperature of 37°C with 5% CO_2_. To develop spheroids, Caco‐2 (ATCC) or HCT8 (ATCC) cells were grown in a cell repellent 96‐well plate (BIOFLOAT 96‐well Cell Culture Plate [F202003]) using Cancer Stem Premium (#20141‐500) supplemented with 1% penicillin/streptomycin and non‐essential amino acids.

### 2.9 Intestinal Organoids Culture

Colon intestinal organoids were purchased from MEMD Millipore, an affiliate of Merck KGaA (14‐881CR) SCC310. Matrigel matrix basement membrane (Corning, #356234) was used for the preparation of the dome. Human basal medium (IntestiCult OGM, #100‐0150) and organoids supplement (#100‐0151) were purchased from Stem Cell Technology and used as medium for the maintenance of organoid culture. Gentle cell dissociation reagent (#100‐0485, Stem Cell Technology organoids) was used to dissociate organoids from the dome. Organoids (*n* = 20–30) were exposed to OMVs (50 µg/mL) in Cancer Stem media supplemented with 1% Matrigel for 18 h.

### 2.10 Plasmids and Transfection

GFP‐Lamp1 (#C10596, Lysosomal marker), GFP‐ER (#C10590, Endoplasmic reticulum marker), and GFP‐Golgi (#C10592, Golgi marker) were used for transient transfection in accordance with the guidelines provided by the manufacturer (Thermo Fisher Scientific). GFP‐Cav1 was a gift from Dr. Ari Helenius (Addgene plasmid #14433; http://n2t.net/addgene:14433; RRID:Addgene_14433). The GFP‐Cav1 plasmid was introduced into HCT8 colon cancer cells using TransIT‐X2 transfection reagent (#MIR 6004) according to the manufacturer's protocol (Mirus Bio LLC, Madison, WI, USA). Subsequently, transfected cells were used for co‐localisation experiment of PKH26‐labelled OMVs (20 µg/mL) with GFP‐Cav1, using live cell confocal microscopy.

### 2.11 Antibodies and Cell Trackers

Anti‐Zo‐1 (#610967, WB = 1:1000, IF = 1:100), Jam‐1 (#612120, WB = 1:1000), β‐catenin (#610153, WB = 1:1000, IF = 1:100), Occludin (#611091, WB = 1:1000, IF = 1:100), antibodies were purchased from BD Bioscience. Anti‐Histone H3 (1G1) (#517576, WB = 1:1000) was purchased from Santa Cruz Biotechnology, β‐actin (#A2066, WB = 1:1000, IF = 1:100) was purchased from Sigma–Aldrich. Anti‐Claudin‐1 (#13050.1‐AP, WB = 1:1000, IF = 1:100) was purchased from Protein Tech, and Anti‐hE‐Cadherin (#MAB18381, WB = 1:1000) was purchased from RD Systems. Alexa Fluor 488/555/647 anti‐Rabbit (IgG) and anti‐mouse (IgG) conjugated secondary antibodies (1:200 dilution) for immunofluorescence were purchased from Thermo Fisher Scientific. Mitotracker green (FM#9074) and Lysotracker green (DND‐26 #8783) were purchased from Cell Signalling. Cell Light Early Endosome (Ref#C10586), Cell Light Golgi‐GFP (Ref#C10592) and Cell Light ER‐GFP(Ref#C10590) were purchased from Thermo Fisher Scientific.

### 2.12 SDS‐PAGE and Western Blot Analysis of OMVs

For the detection of OMV‐associated HapA, the protein content of OMVs in samples isolated from the *V. cholerae* strain A1552 and its isogenic Δ*hapA* mutant was precipitated with 25% trichloroacetic acid (TCA) for 30 min on ice, followed by centrifugation at 21,000 × *g* for 30 min at 4°C. The resulting protein pellets were resuspended in 50 µL of 1× sample buffer containing 10% glycerol, 0.05% bromophenol blue, 2% SDS, 5% 2‐mercaptoethanol and 10 mM Tris‐HCl, pH 6.8. The sample was heat‐treated in a boiling‐water bath for 5 min, then loaded on SDS‐PAGE and transferred onto a nitrocellulose membrane. The membrane was blocked with 5% skimmed milk in phosphate‐buffered saline containing Tween 20 (PBST) at room temperature for 1 h. Following blocking, the membranes were incubated overnight at 4°C with anti‐HapA polyclonal antiserum or anti‐OmpU polyclonal antiserum, both diluted 1:3000 in PBST. Subsequently, the membranes were rinsed three times for 10 min each with PBST, followed by incubation with appropriate HRP‐conjugated secondary antibodies in PBST at room temperature for 1 h. Imaging was performed using LAS‐4000 software on a LAS‐4000 imager (GE Healthcare) with either ECL Bio‐Rad Clarity Western substrate or Super Signal West Femto Maximum Sensitivity Substrate (#34096).

### 2.13 Western Blot Analysis of Mammalian Cells

HCT8 and Caco‐2 cells were seeded in 6‐well plates (Thermo Fisher Scientific) at a density of 3 × 10⁵ cells per well. To achieve monolayer growth, the cells were grown overnight, while polarised growth was attained by culturing the cells for 21 days. For spheroid growth, cells were seeded at 105 cells per well in appropriate spheroid‐promoting conditions and allowed to grow for 4–5 days. Nonpolarised HCT8 cells were treated with increasing concentrations of OMVs or purified HapA for 4 h. The polarised Caco‐2 cells were exposed to OMVs (25 µg/mL) for 4 h. After the treatment, both nonpolarised and polarised cells were lysed with NP40 cell lysis buffer containing protease and phosphatase inhibitors, followed by Western blot analysis. For cell lysis, cells were washed with ice‐cold 1 × PBS and lysed using ice‐cold lysis buffer containing 20 mM Tris‐HCl pH 8, 300 mM KCl, 10% glycerol, 0.25% Nonidet P‐40, 0.5 mM EDTA, 0.5 mM EGTA, 1 mM PMSF, 1× complete protease inhibitor (Roche) and phosSTOP (Roche). The cell lysates were normalised to equal concentrations using the Pierce BCA protein assay kit (#23225, Thermo Scientific) according to the manufacturer's protocol. The lysates were mixed with 4× sample buffer and boiled for 10 min. After that, proteins in the lysates were separated using SDS‐PAGE and transferred onto a nitrocellulose membrane. The membrane was blocked with 5% skimmed milk in PBST for 1 h at room temperature. Following blocking, the membrane was incubated overnight at 4°C with primary antibodies diluted in 5% skimmed milk. The membrane was rinsed three times with PBST (0.1%) for 10 min each, followed by incubation with HRP‐conjugated secondary antibodies in blocking buffer containing 5% skimmed milk for 1 h at room temperature. Protein bands were detected using a ChemiDoc imaging system and visualised using a LAS‐4000 software on a LAS‐4000 imager (GE Healthcare, Chalfont St Giles, UK) by the application of a chemiluminescence reagent ECL BIO‐RAD Clarity Western substrate (Bio‐Rad Laboratories, Inc., USA (Bio‐Rad)) or Super Signal West Femto Maximum Sensitivity Substrate (#34096).

### 2.14 Confocal Microscopy

For the preparation of cells for confocal microscopy, the nonpolarised and polarised epithelial cells were seeded in an 8‐well chamber slide with a coverslip bottom (μ‐Slide, ibidi). The intestinal organoids were seeded in a 24‐well plate. Both 2D and 3D cells were treated with OMVs for the durations specified in the figure legends. After treatment, cells were fixed with 4% paraformaldehyde for 30 min at room temperature, followed by permeabilisation with Triton X‐100 (0.25% in PBS). After permeabilisation, cells were incubated with primary antibodies overnight at 4°C. The next day, cells were incubated with Alexa‐488, ‐555 or ‐647 labelled secondary anti‐rabbit or anti‐mouse antibodies for 1 h at room temperature. After washing with PBS, nuclei were stained with DAPI for 5 min at room temperature. Images were acquired using a Leica SP8 inverted confocal system (Leica Microsystems) equipped with an HC PL APO 63×/1.40 oil immersion lens. Fluorescence intensity profiles were generated using the plot profile function in ImageJ, and image processing was performed using the ImageJ–FIJI distribution (Schindelin et al. 2012).

For live‐cell confocal microscopy, cells were seeded on a coverslip bottom 18‐well chamber slide (μ‐Slide, ibidi) and incubated overnight at 37°C with 5% CO_2_. The next day, cells were transfected with various organelle markers: Cell Light Early Endosome (Ref#C10586), Cell Light Golgi‐GFP (Ref#C10592), Cell Light ER‐GFP(Ref#C10590) or GFP‐Cav1, and incubated overnight at 37°C in 5% CO_2_. After transfection, cells were treated with PKH26‐labelled OMVs (20 µg/mL) for 4 h, followed by co‐staining with nuclear marker Hoechst 33342 (Thermo Fisher Scientific), mitochondrial marker Mitotracker (Thermo Fisher Scientific) and lysosomal marker Lysotracker according to the manufacturer's instructions. For the co‐localisation experiments, cells were exposed to PKH26‐labelled OMVs and FITC‐Dextran 70 kDa (1 mg/mL), followed by live cell imaging using the Leica SP8 inverted confocal system (Leica Microsystems) equipped with an HC PL APO 63×/1.40 oil immersion lens. Imaging was performed in RPMI media without phenol red (Sigma).

For the detection of OMVs‐associated HapA, OMVs (50 µg) isolated from the *V. cholerae* A1552 and A1552Δ*hapA* mutant strains were incubated with anti‐HapA polyclonal antiserum (1 µg) overnight at 4°C in 10% FCS/PBS. The next day, OMVs were washed with PBS following incubation with Alexa‐488 conjugated secondary antibodies (1:200 dilution in 10% FCS/PBS) for 1 h at room temperature. OMVs were then stained with the lipophilic dye PKH26 for 5 min at room temperature, followed by three washes with PBS. OMVs stained for detection of PKH26 and HapA were mounted onto a glass slide and visualised with the Leica SP8 inverted confocal system (Leica Microsystems) equipped with an HC PL APO 63×/1.40 oil immersion lens. Profiles of fluorescence intensity were generated using the plot profile function integrated into ImageJ. The processing of the images was performed using the ImageJ–FIJI distribution (Schindelin et al. 2012).

### 2.15 PKH26 Labelling of OMVs for Host Cell Interaction Studies

OMVs isolated from *V. cholerae* strains A1552 and A1552Δ*hapA* were fluorescently labelled using the PKH26 Red Fluorescent Cell Linker Kit (Sigma–Aldrich, Merck, #MINI26‐1KT), following the manufacturer's instructions with minor modifications. Briefly, pelleted OMVs (300 µg total protein) were resuspended in 1 mL of Diluent C (provided in the PKH26 kit) and gently mixed by pipetting to obtain a homogenous suspension. Diluent C is a physiological buffer that maintains membrane integrity and is optimised for the efficient incorporation of PKH26 into lipid membranes. To label the OMVs, 4 µL of PKH26 dye was added to the 1 mL OMV suspension and mixed gently by pipetting for 30 s to ensure uniform dye distribution. The mixture was then incubated at room temperature for 5 min. To separate labelled OMVs from unbound dye, the sample was ultracentrifuged at 100,000 × *g* for 30 min at 4°C. After centrifugation, the supernatant and interface layer were carefully removed without disturbing the pellet. The labelled OMV pellet was resuspended in sterile PBS and subjected to three consecutive washing steps by ultracentrifugation at 100,000 × *g* for 30 min at 4°C to eliminate residual unincorporated dye. The final OMV pellet was resuspended in PBS, and the protein concentration of the labelled OMVs was determined using a BCA assay kit (Thermo Scientific), according to the manufacturer's instructions.

### 2.16 Flow Cytometry

The uptake of PKH26‐OMVs by HCT8 cells, with or without the specified inhibitors, was performed by flow cytometry analysis as previously described (Nadeem et al. 2021). Briefly, HCT8 cells were exposed to increasing concentrations of PKH26‐OMVs for 4 h followed by flow cytometry analysis. The endocytic pathways of *V. cholerae* OMVs were investigated by exposing HCT8 cells to PKH26‐OMVs (20 µg/mL) for 4 h in the presence or absence of pharmacological endocytic inhibitors. Cells were pretreated for 30 min with individual endocytic inhibitors: amiloride (1 mM), nocodazole (20 µM), LY294002 (20 µM), chlorpromazine (20 µM), Dynasore (25 µM), chlorpromazine (20 µM), methyl β‐cyclodextrane (5 mM), filipin (5 µg/mL), jasplakinolide (500 nM) and cytochalasin D (5 µM), following flow cytometry analysis. The mean fluorescence intensity (MFI) was utilised to characterise the cellular uptake in the experiment, which focused on living cells. The data were shown as a percentage or directly as the mean fluorescence intensity (MFI) after being normalised in relation to PKH26‐OMVs isolated from the wild‐type *V. cholerae* strain A1552.

### 2.17 Cell Toxicity Assay

For cell toxicity assays, HCT8 cells (1 × 10^3^) were seeded in a 96‐well plate and incubated overnight at 37°C. The following day cells were exposed to individual endocytic inhibitors: amiloride (1 mM), nocodazole (20 µM), LY294002 (20 µM), chlorpromazine (20 µM), Dynasore (25 µM), chlorpromazine (20 µM), methyl β‐cyclodextrane (5 mM), filipin (5 µg/mL), jasplakinolide (500 nM), filipin (5 µg/mL), jasplakinolide (500 nM) or cytochalasin D (5 µM) for 4 h at 37°C. Cell viability was measured using [3‐(4,5‐dimethylthiazol‐2‐yl)‐5‐(3‐carboxymethoxyphenyl)‐2‐(4‐sulfophenyl)‐2H‐tetrazolium, inner salt; MTS] (Promega) cell viability assay, according to the manufacturer's instructions. MTS absorbance (490 nm) was measured on an Infinite M200 microplate reader (Tecan). The data were expressed as a percentage and normalised against DMSO‐treated control cells.

For the OMV‐treated cells, HCT8 (1 × 10^3^) cells were similarly seeded in a 96‐well plate and incubated overnight at 37°C. The following day, cells were exposed to OMVs isolated from the *V. cholerae* strains A1552 or A1552Δ*hapA* mutant for 4 h in serum‐free RPMI 1640 media. After treatment, cells were stained with propidium iodide for 30 min to detect dead cells with compromised cell membranes and Hoechst 33342 to stain the total cell population. Images were acquired with SparkCyto imaging system.

### 2.18 Transmission Electron Microscopy

Negative staining for *V. cholerae* OMVs was performed on glow‐discharged copper grids (300 mesh) coated with a thin carbon film (Ted Pella, Redding, CA). A 3 µL sample was added to the grids, followed by two washes with Milli‐Q (MQ) water. The grids were then stained with 1.5% uranyl acetate solution (EMS, Hatfield, PA) and washed again with MQ water. The grids were examined with a Talos L120C electron microscope operating at 120 kV. Transmission electron micrographs (TEM) were acquired with a Ceta 16 M CCD camera and processed using TEM Image & Analysis software ver. 4.17 (FEI, Eindhoven, The Netherlands).

### 2.19 Statistical Analysis

Data are presented as the mean ± standard deviation from a minimum of two independent experiments. To determine statistical significance between various treatment conditions, we performed a one‐way ANOVA and an unpaired *t*‐test using GraphPad Prism. Significance levels were indicated as follows: *p ≤ 0.05, **p ≤ 0.01 and ‘ns’ denoting non‐significant results.

## 3 Results

### 3.1 Characterisation of HapA‐Associated OMVs From *V. cholerae*

**FIGURE 1:**
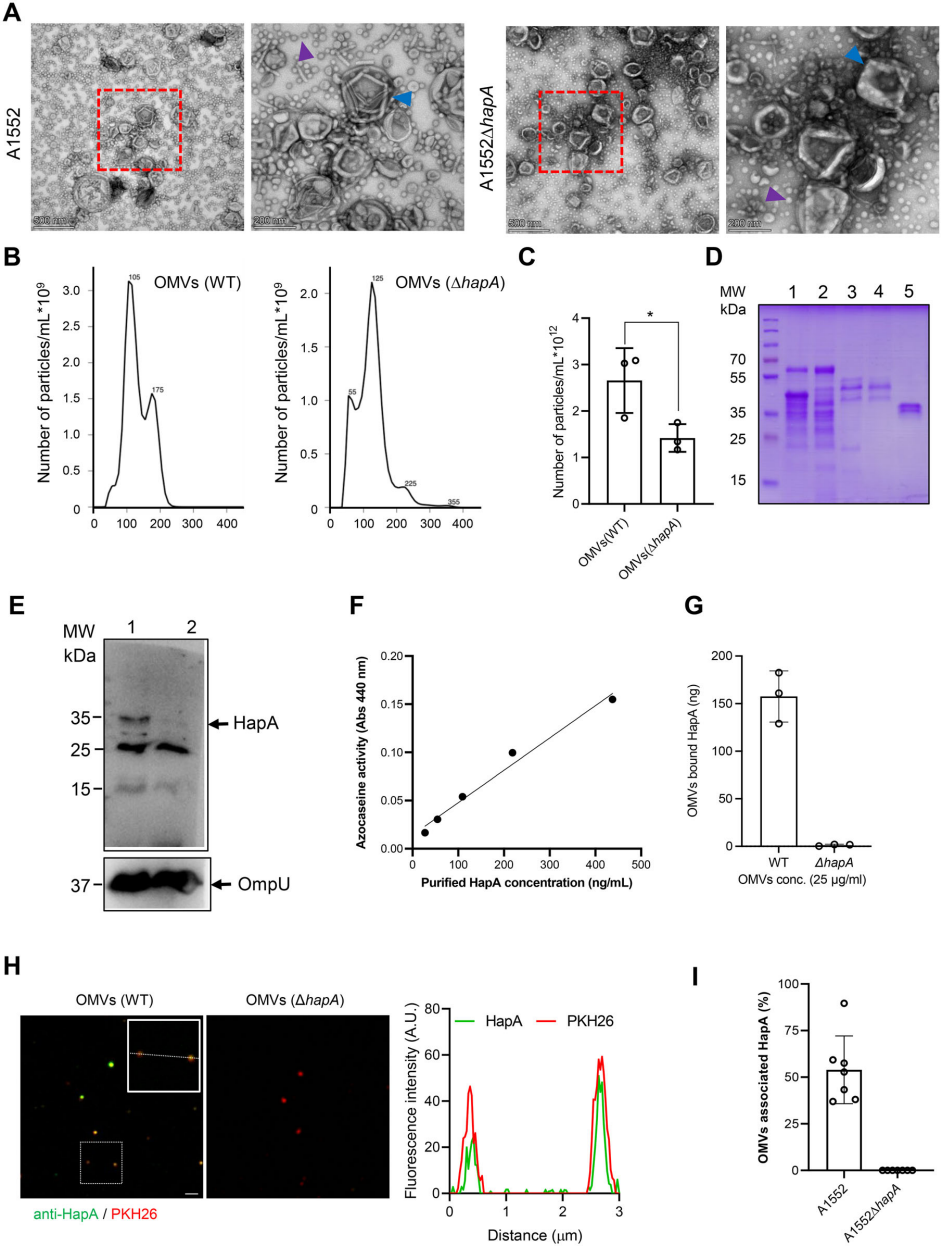
Characterisation of OMVs. (A) Negative staining electron micrograph of purified OMVs isolated from overnight cultures of *V. cholerae* wild‐type strain A1552 and the Δ*hapA* mutant. Large vesicles are indicated by blue arrowheads, while small vesicles are marked with purple arrowheads. Scale bars: 500 and 200 nm. (B) Nanoparticle tracking analysis of OMVs from the wild‐type A1552 strain (left panel) and the Δ*hapA* mutant (right panel). (C) Summary quantification of total data points representing three replicates of nanoparticle tracking analyses, as shown in (B). Data points represent replicates (*n* = 3), and bar graphs show mean ± s.d. Significance was assessed using a non‐parametric *t*‐test. (D) SDS‐PAGE with Coomassie blue staining, illustrating the protein profiles of the OMV samples. Lane 1, culture supernatant of wild‐type *V. cholerae* strain A1552; lane 2, culture supernatant of the Δ*hapA* mutant; lane 3, OMVs from the wild‐type *V. cholerae* strain A1552; lane 4, OMVs from the Δ*hapA* mutant; and lane 5, purified HapA protein. (E) Immunoblot analysis of OMV samples using anti‐HapA polyclonal antiserum (upper panel) Lane 1: OMVs from the wild‐type A1552 strain; Lane 2: OMVs from the Δ*hapA* mutant. The lower panel shows immunoblot analysis using anti‐OmpU polyclonal antiserum as a loading control for OMVs. (F) Azocasein activity assay with different HapA concentrations to obtain a standard curve for estimating the amount of HapA in OMVs. (G) Histogram showing estimated HapA levels associated with OMVs (*n* = 3). (H) Confocal microscopy of OMVs isolated from an overnight culture of *V. cholerae* strain, A1552 WT (left image) and the Δ*hapA* mutant (right image). HapA was stained with anti‐HapA polyclonal antibodies (green), and OMVs were stained with PKH26 lipophilic dye (red). Scale bars: 1 µm. The line graph in the inset indicates where fluorescence intensity profiles were obtained along the white dotted lines, resulting in the corresponding line plot shown on the right. (I) Histogram quantifying the fraction of HapA‐positive OMVs. Data points correspond to the number of images analysed for quantifying HapA‐positive OMVs.

*V. cholerae* produces and secretes a range of toxins and enzymes into the extracellular environment (Kaper et al. 1995). It has been reported that cholera‐causing *V. cholerae* strains release numerous virulence factors, including HapA‐associated outer membrane vesicles (OMVs), during their normal growth (Bitar et al. 2019; Elluri et al. 2014; Mondal et al. 2016; Rompikuntal et al. 2015). To characterise OMV‐associated HapA, we isolated OMVs from both the wild type and *ΔhapA* mutant strains of *V. cholerae* A1552. Transmission electron microscopy (TEM) revealed two distinct populations of OMVs in each strain, with a diameter range of 20–200 nm. Large and small vesicles are indicated by blue and purple arrowheads, respectively (Figure 1A). To further analyse the size distribution of OMVs, we performed nanoparticle tracking analysis (Nanosight), which revealed that the OMV samples were heterogeneous in size. For OMVs isolated from the wild type A1552 strain, diameters ranged from 20 to 200 nm, with two predominant populations at approximately 105 and 175 nm. Similarly, OMVs from the ∆*hapA* mutant strain exhibited size heterogeneity, with diameters ranging from 20 to 355 nm, and were distributed across three major peaks at 55, 125 and 255 nm (Figure 1B,C). To visualise the major protein components of OMVs, SDS‐PAGE analysis was performed. A comparison between OMVs from the wild‐type *V. cholerae* strain A1551 and its isogenic Δ*hapA* mutant revealed a similarity in the major protein bands, as well as in most of the minor bands (Figure 1D). To assess whether HapA was associated with the OMVs, immunoblot analysis was conducted using anti‐HapA polyclonal antisera. OMVs from the ∆*hapA* mutant served as a negative control. Immunoblot analysis confirmed the presence of 35 KDa HapA in OMV preparations from the wild‐type strain. Protein loading in the SDS‐PAGE gel was normalised to 3 µg per well, and the immunoblot band detected with anti‐OmpU antiserum was used as a marker for OMVs and as a loading control (Figure 1E). Both TEM and NTA revealed the presence of heterogeneous OMV populations, with TEM detecting vesicles in the 20–200 nm range and NTA identifying two predominant peaks at approximately 105 and 175 nm in OMVs from the wild‐type strain (Figure 1A). To determine which vesicle population is more abundantly associated with HapA, OptiPrep density gradient centrifugation was performed to separate the large and small vesicles (Figure S1A). Although complete separation was not achieved, electron microscopy confirmed that specific fractions were enriched in either large or small vesicles (Figure S1B). Immunoblot analysis of these fractions using anti‐HapA antiserum demonstrated that larger vesicle fractions contained higher levels of HapA (Figure S1C). The outer membrane protein OmpA was used as a marker to identify OMV‐containing fractions and served as a loading control. Notably, immunoblot analysis using anti‐OmpA antiserum detected OmpA in both large and small vesicle‐enriched fractions (Figure S1C). This finding indicates that the smaller vesicle population is not merely composed of lipid particles but instead is derived from the bacterial outer membrane. Therefore, both the large and small vesicle populations are likely bona fide outer membrane vesicles.

To evaluate the enzymatic activity of OMV‐associated HapA and quantify its concentration, we performed an Azocasein enzyme assay following the protocol detailed in the Section 2. The enzymatic activity of purified HapA was used to generate a standard curve for correlating HapA concentration with its activity (Figure 1F). Based on the results, we determined that approximately 150 ng of HapA is associated with 25 µg/mL of OMVs (Figure 1G). The enzymatic activity of OMVs was also examined by their ability to degrade proteins on skimmed milk agar plates (Figure S2A). To further confirm the association of HapA with OMVs, we utilised confocal microscopy to detect HapA on OMVs, using OMVs from the Δ*hapA* strain as a control. This analysis verified the association of HapA with OMVs (Figure 1H,I). These findings confirm that the active form of HapA secreted by *V. cholerae* is associated with OMVs and that OMV‐associated HapA exhibits enzymatic activity. Additionally, to analyse the surface chemical composition of wild‐type and Δ*hapA* mutant OMVs, we employed cryo‐XPS analysis. In wild‐type OMVs, the surface carbon (C) atoms were distributed as follows: 11 ± 2% in lipids, 64 ± 2% in proteins (or peptidoglycan) and 25 ± 0.5% in polysaccharides. The Δ*hapA* mutant OMVs exhibited a distribution of 30 ± 0.5% in lipids, 61 ± 0.5% in proteins (or peptidoglycan) and 9 ± 0.0% in polysaccharides. These results indicate an increase in lipid content and a decrease in the relative amount of surface polysaccharides in the mutant OMVs.

### 3.2 OMV‐Associated HapA Induces Detachment of Intestinal Epithelial Cells in a Time‐Dependent Manner

**FIGURE 2:**
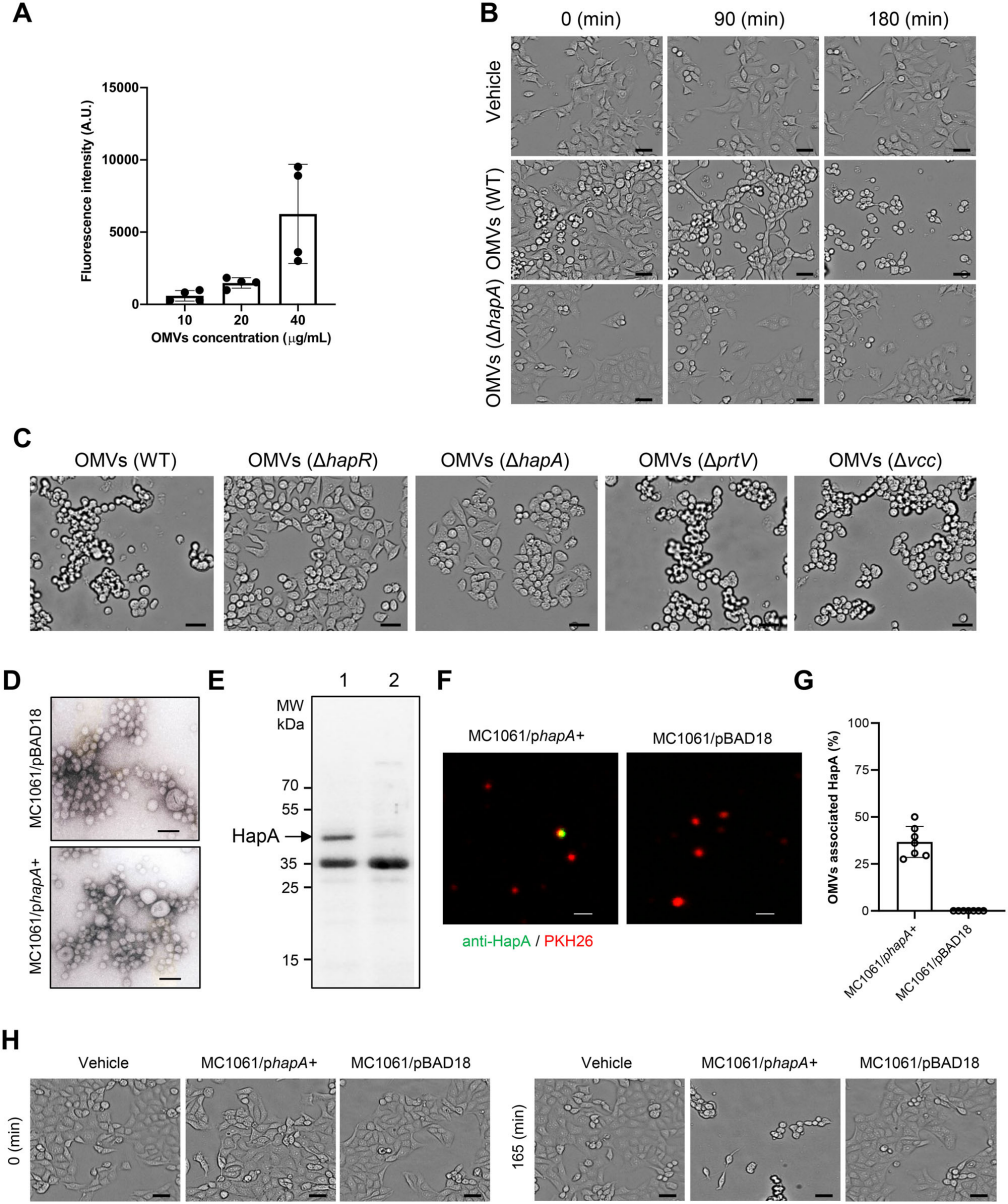
OMV‐associated HapA causes rounding of the epithelial cells. (A) Flow cytometry analysis showing concentration‐dependent uptake of PKH26‐labeled OMVs by epithelial cells. Data points represent replicates (*n* = 4); bar graphs show mean ± s.d. (B) Live imaging of Caco‐2 cells exposed to OMVs (25 µg/mL) or vehicle control (PBS) using the SparkCyto imaging system. *V. cholerae* OMVs induce time‐dependent rounding and detachment of HCT8 cells. Scale bars, 20 µm. (C) HCT8 cells were exposed to OMVs (25 µg/mL, 4 h) isolated from wild‐type *V. cholerae* strain A1552 and its isogenic mutants (Δ*hapR*, Δ*hapA*, Δ*prtV* and Δ*vcc*). Images were acquired live using SparkCyto imaging system. Scale bars: 20 µm. (D) Negative staining electron micrographs of purified OMVs isolated from overnight cultures of *E. coli* strain MC1061 harbouring the empty plasmid vector pBAD18 (upper panel: MC1061/pBAD18) and the HapA expressing construct (lower panel: MC1061/p*hapA* ^+^). Scale bars: 150 nm. (E) Immunoblot analysis of OMV samples using anti‐HapA polyclonal antiserum. Lane 1: OMVs from strain MC1061/p*hapA* ^+^, Lane 2: OMVs from strain MC1061/pBAD18. (F and G) Confocal microscopy of OMVs isolated from overnight cultures of *E. coli* strains MC1061/p*hapA*+ (left panel) and MC1061/pBAD18 (right panel). OMVs were stained with PKH26‐labelled lipophilic dye (red) and HapA‐specific polyclonal antibodies (green). Scale bars, 1 µm. The histogram shows the quantification of HapA‐positive OMVs. The data points correspond to the number of images used for quantification of HapA‐positive OMVs. (H) Caco‐2 cells were exposed to OMVs isolated from the overnight growth culture of *E. coli* strain MC1061/pBAD18 or MC1061/p*hapA* ^+^. Scale bars, 20 µm.

Previous studies have demonstrated that HapA, when associated with OMVs, can induce apoptosis in Int407 cells in a dose‐dependent manner (Mondal et al. 2016). However, it is important to consider that Int407 cells are derived from the HeLa cervical cancer cell line (Gartler 1968), which could potentially influence the results, given that HapA is a virulence factor of *V. cholerae* known for causing gastrointestinal infections. Therefore, to ensure a more relevant experimental context, we utilised commercially available gut‐derived cell lines, such as HCT8 and Caco‐2 cell lines. These cell lines are powerful tools for investigating the molecular mechanisms underlying intestinal function in response to *V. cholerae* infection (Krebs and Taylor 2011; Mel et al. 2000; Nadeem et al. 2021). Upon treating HCT8 cells with increasing concentrations of PKH26‐labelled OMVs (PKH26‐OMVs) for 4 h, flow cytometry analysis indicated a concentration‐dependent uptake of OMVs by the cells. Using an OMV concentration of 20 µg/mL resulted in moderate OMV entry into the cells (Figure 2A). Based on this observation, we selected this concentration for further experiments to analyse the interaction between OMVs and cells using confocal microscopy.

To investigate the impact of OMV‐associated HapA on epithelial cells, we treated Caco‐2 cells with OMVs derived from either the wild‐type or Δ*hapA V. cholerae* strains. We observed that cell detachment and rounding were dependent on the presence of HapA. Notably, OMVs from the wild‐type *V. cholerae* A1552 strain caused significant changes in cell morphology, leading to time‐dependent cell detachment, whereas OMVs from the Δ*hapA* mutant did not induce these effects (Figure 2B). Importantly, treatment with OMVs from both the wild‐type and Δ*hapA* mutant strains did not cause any detectable toxicity in the epithelial cells (Figure S2B). Furthermore, we examined the effects of other virulence factors associated with OMVs from *V. cholerae*. The *V. cholerae* protease PrtV (a virulence factor regulated by a quorum sensing regulator, HapR) (Vaitkevicius et al. 2006) and *V. cholerae* cytolysin (VCC, a pore‐forming toxin whose function is affected by HapR) (Van der Henst et al. 2018) have been shown to be released in association with OMVs (Elluri et al. 2014; Rompikuntal et al. 2015). We investigated the effects of OMV‐associated PrtV and VCC on HCT8 cells. Microscopic examination with the SparkCyto imaging system revealed that changes in the morphology of HCT8 cells were affected by treatment with OMVs isolated from the wild‐type A1552 strain, as well as the *prtV* and *vcc* deletion mutants. However, no similar effect was observed with OMVs from the Δ*hapR* and Δ*hapA* mutants (Figure 2C). In addition to PrtV and HapA, previous studies have identified the production of VesA, VesB and VesC proteases by *V. cholerae* (Sikora et al. 2011). To eliminate the potential effects of other proteases and secreted toxins from *V. cholerae*, HapA was expressed in the commensal *E. coli* strain MC1061. OMVs were isolated from both the HapA‐producing (MC1061/p*hapA*+) strain and vector control (MC1061/pBAD18). To examine the ultrastructure of OMVs from both strains, TEM analysis was performed, revealing similar morphologies of OMVs from the two strains (Figure 2D). The protein profiles of OMVs isolated from the HapA‐producing strain and the vector control strain were subjected to SDS‐PAGE and Coomassie blue staining, and immunoblot analysis (Figure S2C). The presence of HapA was confirmed in OMVs from the MC1061/p*hapA* ^+^ strain, as indicated by the detection of the 45 kDa band, which was absent in the vector control (Figure 2E). Notably, the OMV‐associated form of HapA in *E. coli* MC1061/p*hapA*+ was detected as the non‐processed 45 kDa form. In contrast, OMVs isolated from the wild‐type *V. cholerae* strain A1552 contained a processed 35 kDa form of HapA. Additionally, confocal microscopy verified the association of HapA with the OMVs (Figure 2F,G). Consistent with the effects observed with OMVs from the wild‐type *V. cholerae* strain, OMVs derived from the HapA‐expressing *E. coli* strain MC1061/p*hapA*+ also induced detachment of Caco‐2 cells (Figure 2H). These results collectively confirm that OMV‐associated HapA is biologically active.

### 3.3 *V. cholerae* OMVs Exhibit Rapid Internalisation Into the Endolysosomal Compartment of Intestinal Epithelial Cells

The internalisation of OMVs by epithelial cells plays a crucial role in host‐microbe interactions (Zlatkov et al. 2021), facilitating the direct translocation of bacterial components into host cells. To explore this process, we conducted a detailed analysis of OMV uptake by HCT8 cells using both flow cytometry and confocal microscopy to elucidate their cellular interactions.

**FIGURE 3:**
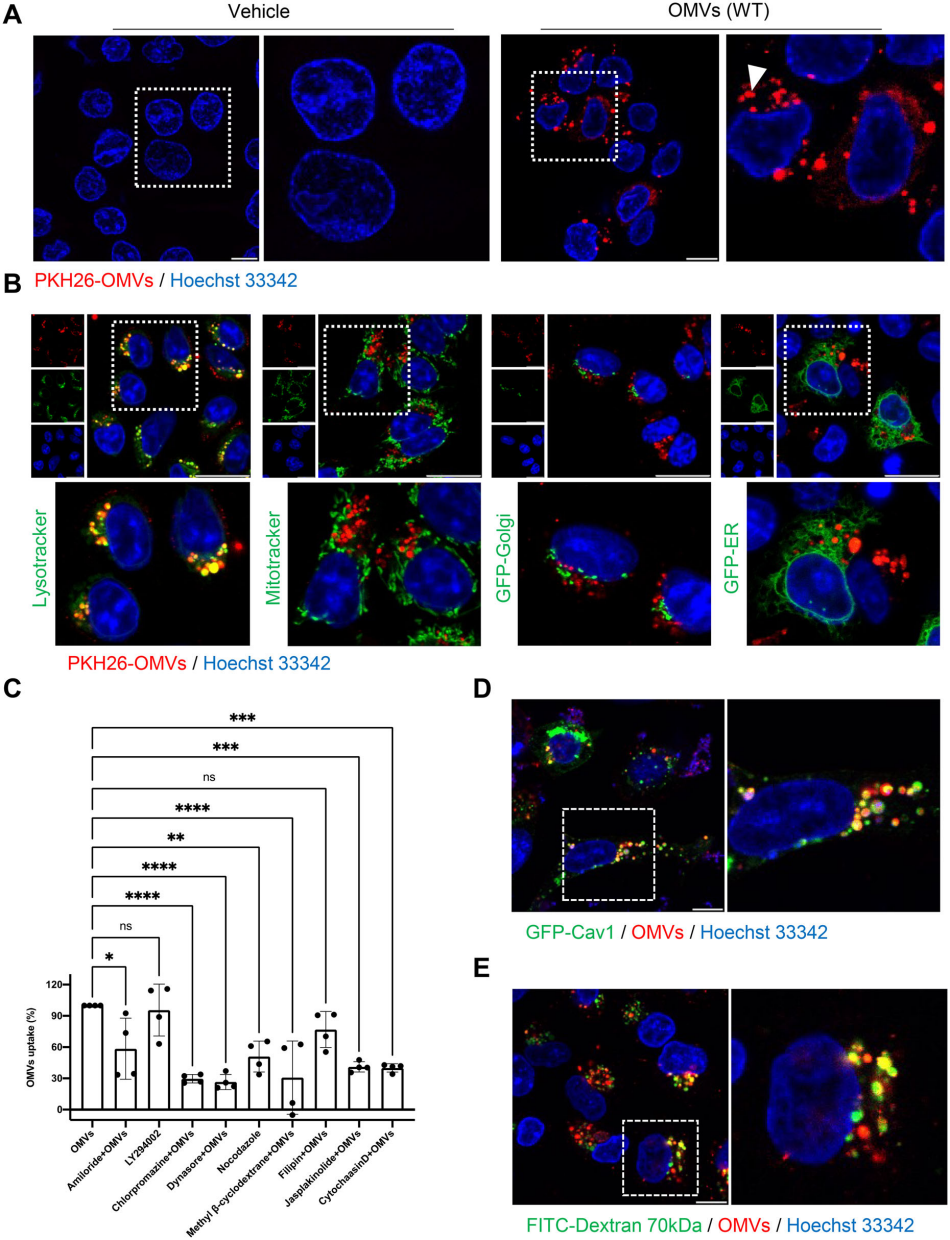
*V. cholerae* OMVs enter epithelial cells through clathrin‐mediated endocytosis, caveolin‐mediated endocytosis and macropinocytosis, and accumulate in the endolysosomal compartment. (A) HCT8 cells were treated with PKH26‐labelled OMVs (20 µg/mL) for 4 h, and cellular distribution of OMVs (red) was visualised by confocal microscopy. The nuclei were counterstained with Hoechst 33342 (blue). OMVs are mainly localised in the cytosolic vesicular compartment. (B) Co‐localisation of PKH26‐labelled OMVs (red) with cellular markers (green) in HCT8 cells treated with OMVs (20 µg/mL) for 4 h. The nuclei were counterstained with Hoechst 33342 (blue). Scale bars: 10 µm. (C) HCT8 cells were treated with PKH26‐OMVs (20 µg/mL) for 4 h, with or without endocytosis inhibitors: Amiloride (1 mM), LY294002 (20 µM), chlorpromazine (20 µM), Dynasore (50 µM), nocodazole (20 µM), methyl β‐cyclodextrane (5 mM), filipin (5 µg/mL), jasplakinolide (500 nM) or cytochalasinD (2 µM). Inhibitors were added 30 min prior to the addition of PKH26‐OMVs. Cellular uptake of OMVs was quantified by flow cytometry. Data points represent four replicates from two biologically independent experiments; bar graphs show mean ± s.d. Statistical significance was determined using ANOVA with Dunnett's post‐test, comparing inhibitor‐treated samples to the OMV‐only control. *p ≤ 0.05, **p ≤ 0.01, ns = not significant. (D) HCT8 cells were transfected with GFP‐Cav1 for 24 h and subsequently treated with PKH26‐OMVs (20 µg/mL) for 4 h. The nuclei were counterstained with Hoechst 33342 (blue). (E) HCT8 cells were treated with FITC‐70 kDa dextran (1 mg/mL) and PKH26‐OMVs (20 µg/mL) for 4 h. The nuclei were counterstained with Hoechst 33342. Scale bars, 10 µm.

We observed that OMVs were predominantly localised within the cytosolic vesicular compartment of the cell, with notable accumulation in the perinuclear region (Figure 3A). We compared the entry efficiency of OMVs from the Δ*hapA* mutant with that of the wild‐type strain into host cells. A reduction in entry was observed for OMVs derived from the Δ*hapA* mutant (Figure S2D,E). This decrease in entry efficiency may be attributed to the lower carbohydrate content of OMVs from the Δ*hapA* mutant, as revealed by the XPS assay. Previous research has shown that the polysaccharide structure of OMVs is a key factor influencing the entry kinetics of bacterial membrane vesicles into host cells (O'Donoghue et al. 2017). To further investigate the fate of OMVs within epithelial cells, we transfected HCT8 cells overnight with GFP‐tagged markers for lysosomes (GFP‐LAMP1), endoplasmic reticulum (GFP‐calreticulin‐KDEL) or Golgi apparatus (GFP‐N‐acetyl galactosaminyl transferase), followed by treatment with PKH26‐OMVs (20 µg/mL, 4 h). Live cell confocal microscopy revealed that the majority of OMVs located in the perinuclear region accumulate within the lysosomes, as indicated by the co‐localisation of PKH26‐OMVs with both Lysotracker, a fluorescent dye commonly used to label lysosomes, and GFP‐LAMP1, a marker specifically targeting lysosomes (Figures 3B and S2F). Conversely, no co‐localisation was observed with the markers for the endoplasmic reticulum, Golgi apparatus or mitochondria (Mitotracker) (Figure 3B). These findings collectively suggest a preferential targeting of *V. cholerae* OMVs to the lysosomal compartment within epithelial cells.

### 3.4 The Internalisation of *V. cholerae* OMVs Into Target Host Cells Relies on Caveolin‐, Clathrin‐ and Macropinocytosis‐Dependent Pathways

Endocytic processes of bacterial vesicles are vital for delivering extracellular cargo into the intracellular compartments of mammalian cells, initiating cell signalling crucial for host‐pathogen interactions (O'Donoghue and Krachler 2016). To determine the specific endocytic pathways involved in the cellular uptake of OMVs secreted by *V. cholerae*, we employed a range of pharmacological inhibitors targeting the three major endocytic pathways: macropinocytosis, clathrin‐mediated endocytosis and lipid raft/caveolin‐dependent endocytosis. HCT8 cells were pre‐treated with these inhibitors for 30 min, followed by exposure to PKH26‐OMVs (20 µg/mL, 4 h), and OMV uptake was quantified using flow cytometry. Results revealed that amiloride, an inhibitor of Na+/H+ exchange and macropinocytosis, partially blocked OMV uptake. However, LY294002, a PI3K inhibitor known to affect macropinocytosis, did not inhibit OMV uptake, suggesting that PI3K activity is not required for the cellular uptake of OMVs (Figure 3C). Both chlorpromazine, an inhibitor of clathrin‐mediated endocytosis, and dynasore, an inhibitor of dynamin, exhibited significant reductions in OMV uptake. Importantly, both inhibitors demonstrated similar levels of inhibition (Figure 3C). Regarding lipid raft/caveolin‐mediated endocytosis, methyl‐β‐cyclodextrin, a cholesterol‐depleting agent, decreased OMV uptake, while filipin, a cholesterol‐stabilising molecule, had no significant effect (Figure 3C). Cell viability was assessed in response to the effects of cellular uptake inhibitors, and no significant toxicity to the cells was observed (Figure S3A). These findings suggest a complex interplay of multiple endocytic pathways in the cellular uptake of *V. cholerae* OMVs.

The role of the actin cytoskeleton and microtubules in regulating various endocytic processes has been previously documented (Yarar et al. 2005). To investigate the involvement of the actin cytoskeleton in the cellular uptake of OMVs, we utilised the cell‐permeant compounds cytochalasin D and jasplakinolide, known to disrupt intracellular actin dynamics (Fujimoto et al. 2000). Both inhibitors significantly reduced the cellular uptake of OMVs. Similarly, the microtubule‐depolymerising agent nocodazole also markedly decreased OMVs uptake (Figure 3C). The data from flow cytometry was further confirmed by confocal microscopy for selected pharmacological inhibitors such as chlorpromazine, dynasore and amiloride (Figure S3B).

To investigate the role of caveolin in OMV endocytosis, HCT8 cells transfected with GFP‐Cav1 were exposed to PKH26‐OMVs (20 µg/mL) for 4 h, followed by live cell confocal microscopy. Strong co‐localisation was observed between PKH26‐OMVs and GFP‐Cav1, indicating the involvement of caveolin and cholesterol in OMV uptake (Figure 3D). Notably, the OMVs within the caveolin structures displayed dynamic behaviour (Movie S1). To further confirm the involvement of macropinocytosis and clathrin‐dependent endocytosis, we performed co‐localisation experiments. For macropinocytosis, we examined the co‐localisation of PKH26‐OMVs with FITC‐dextran (70 kDa), a macropinocytosis marker. This analysis revealed weak co‐localisation (Figure 3E), aligning with the partial reduction observed with amiloride (Figure 3C).

Overall, these experiments complement the initial findings presented in Figure 2, providing a more comprehensive understanding of the cellular processes involved in the uptake of *V. cholerae* OMVs. By employing a variety of pharmacological inhibitors and conducting co‐localisation studies, we demonstrated the involvement of multiple distinct endocytic pathways in *V. cholerae* OMVs internalisation.

### 3.5 OMV‐Associated HapA Causes Disruption of Tight and Adherence Junctions

**FIGURE 4:**
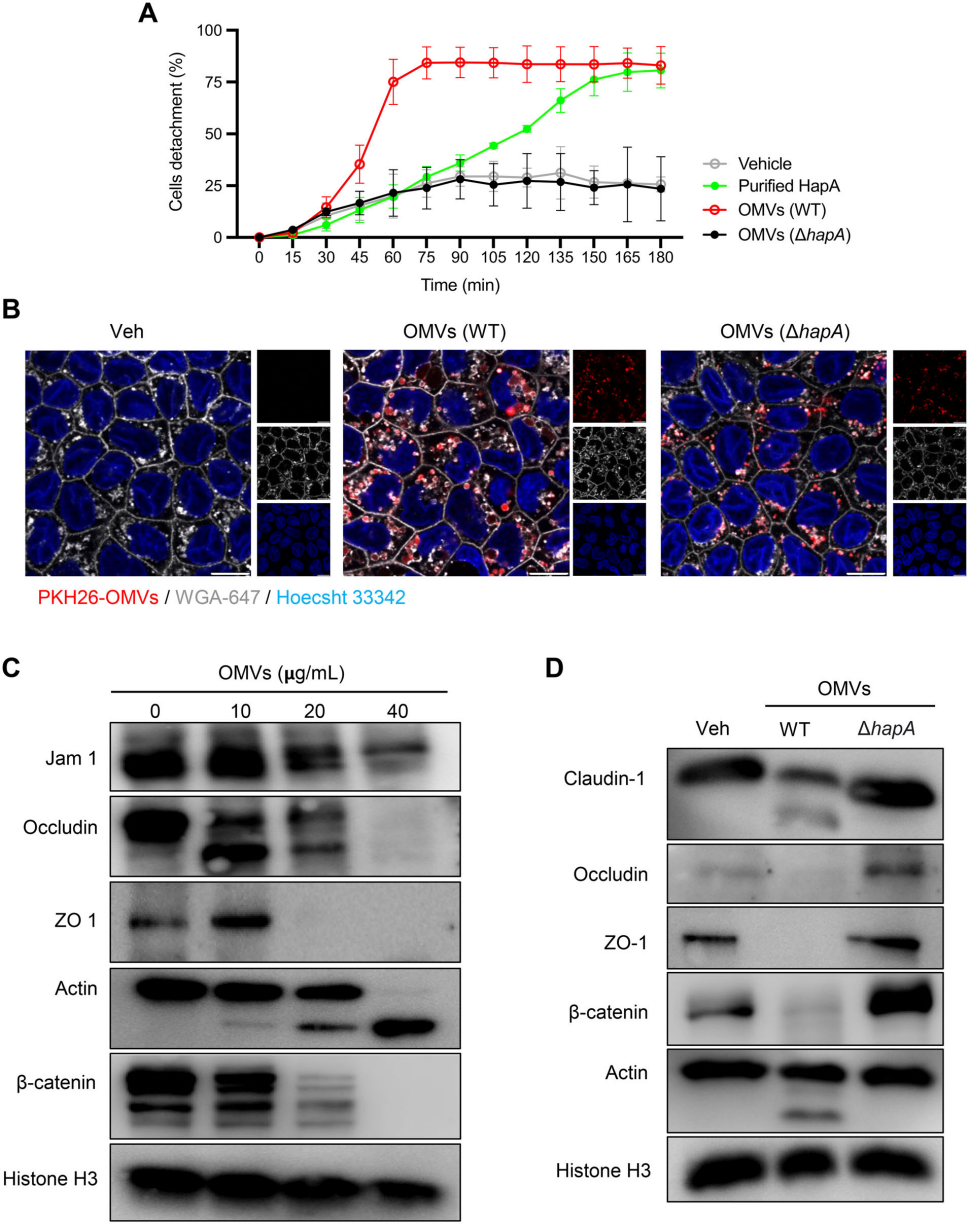
OMV‐associated HapA is biologically more active and causes disruption of tight and adherens junction proteins. (A) Kinetics of Caco‐2 cells detachment in response to purified HapA (5 nM) or OMVs isolated from A1552 wild‐type or the Δ*hapA* mutant (OMVs Δ*hapA*). The line graph indicates the number of detached cells over time. Data points represent replicates (*n* = 3). (B) Polarised Caco‐2 cells were treated with PKH26‐OMVs (25 µg/mL) isolated from A1552 WT or the Δ*hapA* mutant for 4 h. Cellular distribution of OMVs (red) was visualised by confocal microscopy. Nuclei were counterstained with Hoechst 33342 (blue), and cell membranes were labelled with WGA‐647 (grey). OMVs were localised in the cytosolic vesicular compartment. (C) HCT8 nonpolarised cells were exposed to increasing concentrations of OMVs for 4 h, followed by Western blot analysis. *V. cholerae* OMVs caused concentration‐dependent cleavage of tight and adherens junction proteins. Histone H3 was used as a loading control. (D) Polarised Caco‐2 cells were exposed to OMVs (25 µg/mL, 4 h) isolated from A1552 WT or the Δ*hapA* mutant, followed by Western blot analysis. *V. cholerae* OMVs caused cleavage of tight and adherens junction proteins in polarised Caco‐2 cells. Histone H3 was used as a loading control.

**FIGURE 5:**
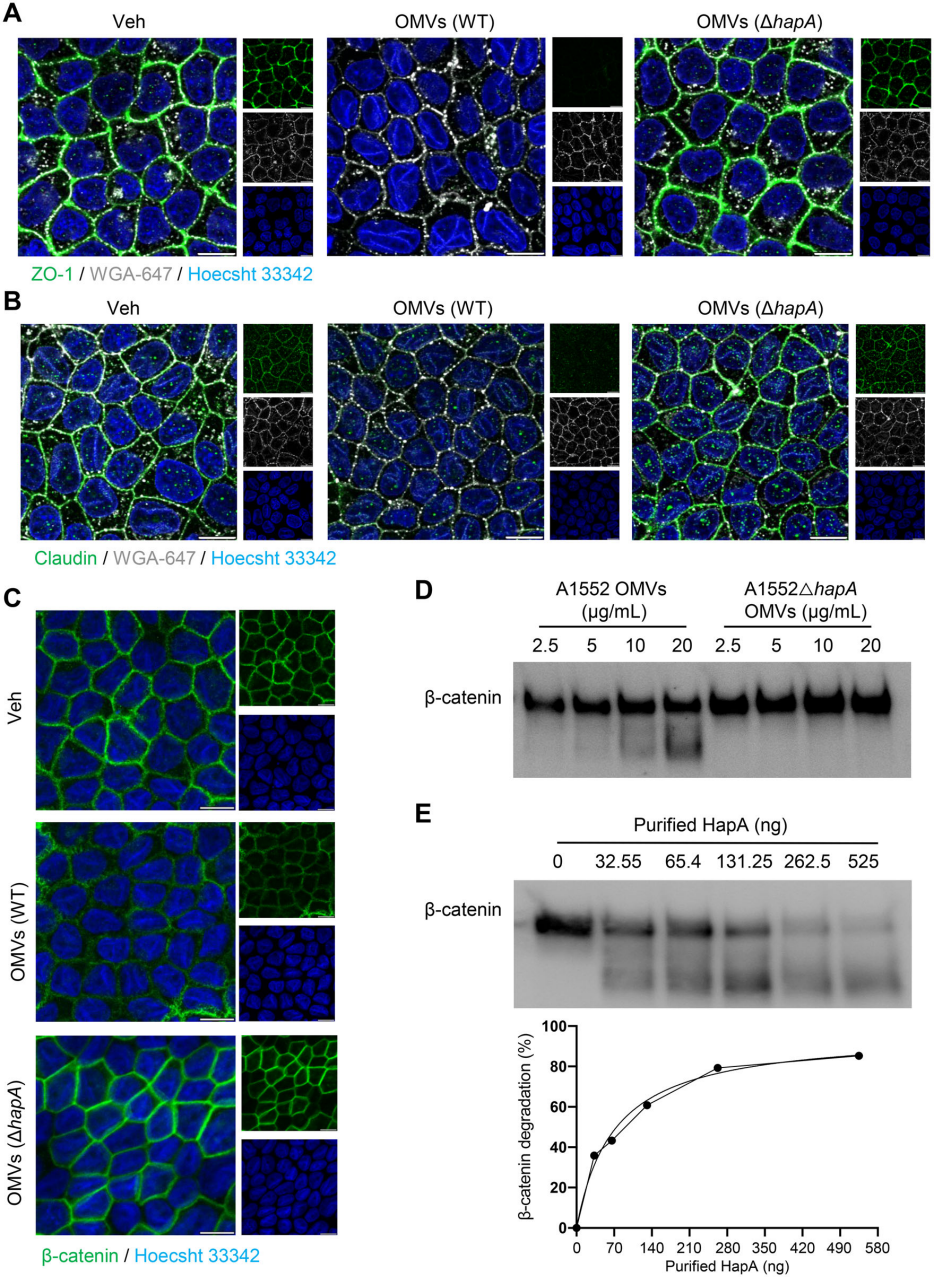
Adherence‐junction protein β‐catenin is a direct substrate of HapA. (A–C) Polarised Caco‐2 cells were treated with *V. cholerae* OMVs (25 µg/mL) isolated from A1552 wild‐type or the Δ*hapA* mutant (OMVs Δ*hapA*) for 4 h, followed by fixation and staining for tight junction proteins ZO‐1 (green) and Claudin (green), as well as the adherens junction protein β‐catenin (green). Cells were counterstained with nuclear marker Hoechst 33342 (blue) and the cell membrane marker WGA‐647 (grey) and visualised by confocal microscopy. OMV‐associated HapA caused a decrease in staining of both tight‐junction proteins (ZO‐1 and Claudin) and the adherens junction protein β‐catenin. (D) Western blot analysis of in vitro reconstitution experiment for purified β‐catenin (100 nM), exposed to increasing concentrations of OMVs isolated from A1552 WT or the Δ*hapA* mutant for 1 h. OMV‐associated HapA caused concentration‐dependent degradation of β‐catenin. (E) Western blot analysis of β‐catenin (100 nM) exposed to increasing concentrations of HapA. The graph indicates concentration‐dependent cleavage of β‐catenin, with best‐fit values generated using the Michaelis‐Menten equation.

Employing the SparkCyto imaging system, we monitored the kinetics of morphological changes in Caco‐2 cells (Figure 4A). Using a deep learning tool, Cellpose, we segmented images of adherent cells at different time points, revealing a time‐dependent detachment of HCT8 cells in response to both purified HapA and OMV‐associated HapA. Notably, OMV‐associated HapA caused a more rapid time‐dependent detachment compared to purified HapA (Figure 4A). The epithelial junctional complex, which includes tight junctions (TJs), adherens junctions (AJs) and desmosomes, plays a critical role in epithelial cell polarity and barrier function against pathogenic molecules (Farquhar and Palade 1963; Ikenouchi et al. 2003). Polarised Caco‐2 cells have been widely used as a model system for studying the impact of bacterial secreted effector molecules on TJs (Benitez and Silva 2016; Farquhar and Palade 1963; Ikenouchi et al. 2003). In our study using polarised Caco‐2 cells, confocal microscopy revealed that the majority of PKH26‐labelled OMVs exposed to these cells were localised either on the cell membrane or within the intracellular compartment (Figure 4B). Interestingly, polarised Caco‐2 cells swiftly internalised OMVs from both the wild‐type and the A1552Δ*hapA* strain (Figure 4B). This suggests, like their uptake in non‐polarised cells (Figures 2A and 3A), that *V. cholerae* OMVs are rapidly internalised by polarised cells (Figure 4B), underscoring a consistent mechanism of OMV‐cell interaction across different cellular contexts. Subsequent Western blot analysis of non‐polarised HCT8 and polarised Caco‐2 cells showed that OMV‐associated HapA caused a dose‐dependent decrease in the expression of TJ and AJ proteins (Figures 4C,D and S4A‐D). This was further supported by confocal microscopy, which showed a significant reduction in the staining of TJ markers, claudin, ZO‐1 and β‐catenin in response to OMV‐associated HapA (Figure 5A‐C). Importantly, this decrease in protein expression was specifically attributed to HapA bound to the OMVs. To explore the possibility of OMV‐associated HapA directly targeting β‐catenin, we conducted in vitro co‐incubation experiments using OMVs derived from wild‐type *V. cholerae* or the Δ*hapA* mutant, alongside purified β‐catenin. A dose‐dependent degradation of β‐catenin was observed when co‐incubated with OMVs from the wild‐type *V. cholerae*. However, this effect was absent when β‐catenin was co‐incubated with OMVs from the Δ*hapA* mutant, indicating a direct interaction between HapA and β‐catenin in the degradation process (Figures 5D and S5A,B). This interaction was further confirmed by co‐incubating purified β‐catenin with purified HapA (Figure 5E). The collective results from our experiments suggest that HapA, associated with *V. cholerae* OMVs, targets both TJ and AJ proteins, indicating that these junctional proteins are substrates for HapA's enzymatic activity. This activity underscores the significant role of HapA in disrupting epithelial cell junctions, thereby contributing to the pathogenicity of *V. cholerae*.

### 3.6 OMV‐Associated HapA Causes Disruption of Tight and Adherence Junctions in 3D Spheroids and Intestinal Organoids

**FIGURE 6:**
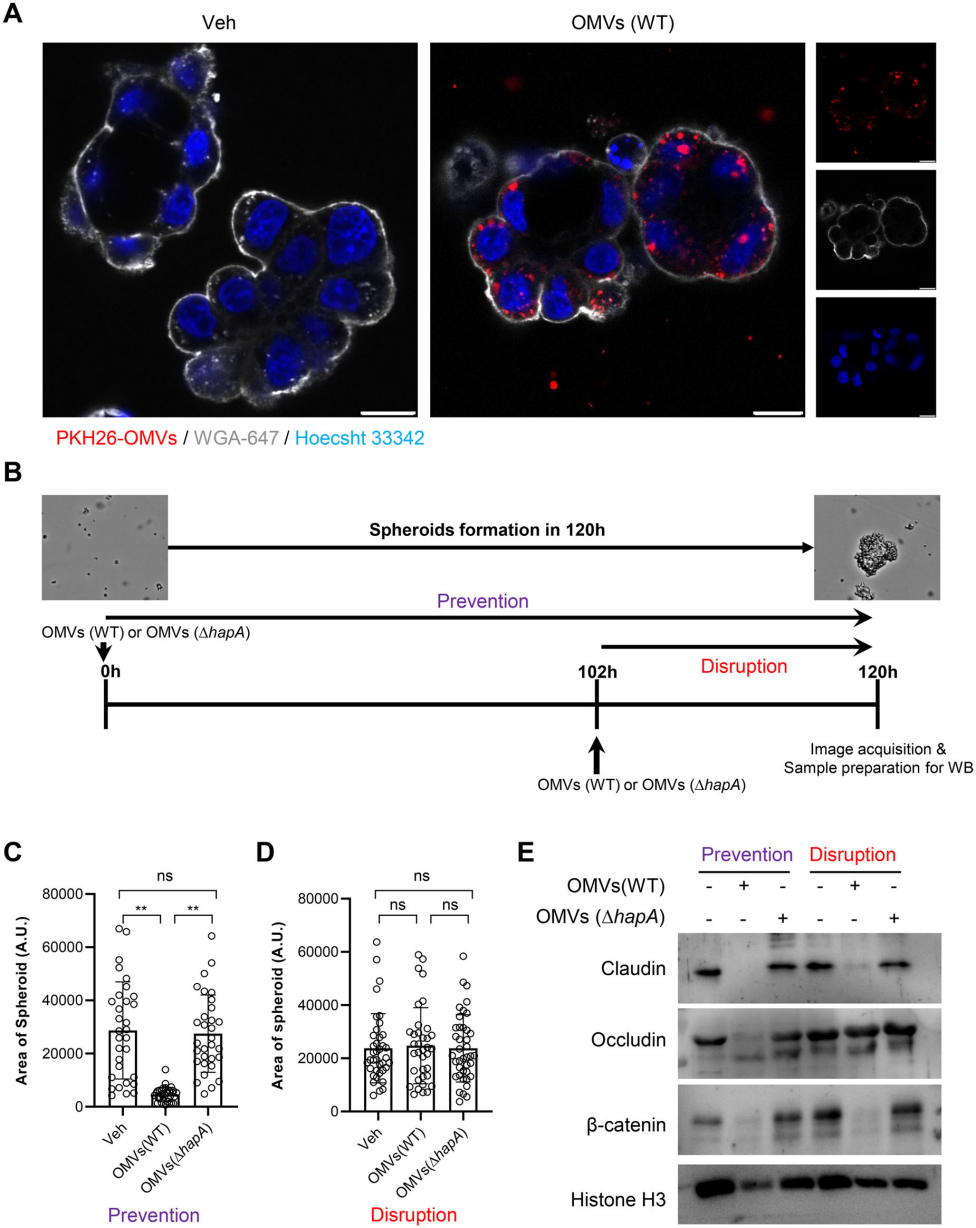
OMV‐associated HapA induces degradation of tight‐ and adherens‐junction proteins in epithelial cells grown in 3D spheroids. (A) Caco‐2 cells grown in 3D spheroids were treated with PKH26‐OMVs (50 µg/mL, 4 h) isolated from *V. cholerae* strain A1552. Confocal microscopy visualised the cellular distribution of PKH26‐labelled OMVs (red). The 3D spheroid cells were counterstained with the nuclear marker, Hoechst 33342 (blue) or the cell membrane marker, WGA‐647 (grey). OMVs were localised in the vesicular compartment of the Caco‐2 cell spheroids. (B) Schematic overview of spheroids exposed to OMVs isolated from A1552 WT or the Δ*hapA* mutant (OMVs Δ*hapA*) for the indicated time periods. (C and D) HCT8 cells were exposed to OMVs isolated from A1552 WT or Δ*hapA*. HapA‐containing OMVs impaired the spheroid formation as quantified by a decrease in spheroid area. Data points in the histogram represent the number of spheroids (30–35) used for area quantification. (E) Spheroids were exposed to OMVs (50 µg/mL) at 102 and 18 h time points followed by Western blot analysis. OMVs from wild‐type A1552 caused cleavage of tight and adherens junction proteins in the spheroids, while OMVs from Δ*hapA* did not. Histone H3 was used as a loading control.

The investigation of cellular responses to OMVs has traditionally been conducted using cell lines (Bermudez‐Brito et al. 2013). However, recent advancements in organoid technology have opened new avenues for exploring the impact of bacterial secreted effector molecules in settings that more accurately reflect in vivo conditions (Dutta and Clevers 2017). Employing 3D cell culture models provides a better understanding of the mechanisms underlying the action of these molecules, closely mimicking in vivo conditions compared to traditional 2D cultures (Kapalczynska et al. 2018). In our investigation, 3D spheroids formed from Caco‐2 cells were exposed to *V. cholerae* OMVs (20 µg/mL, 6 h), revealing OMVs' localisation within the intracellular cytosolic compartment of the spheroids (Figure 6A). A comparison of spheroid formation between Caco‐2 and HCT8 cells indicated that HCT8 cells formed more compact spheroids, prompting further investigation into the effects of OMV‐associated HapA on HCT8 spheroid formation (Figures 6B,D and S6A,B). We specifically focused on evaluating the effects of HapA‐associated OMVs on both the formation and integrity of spheroids, employing two distinct experimental setups: one aimed at preventing spheroid formation using 50 µg/mL of OMVs for 120 h treatment and another at disrupting pre‐formed spheroids, applying 50 µg/mL of OMVs for an 18 h period (Figure 6B). Notably, OMV‐associated HapA inhibited HCT8 spheroid formation, evidenced by reduced spheroid diameter (Figure 6C). However, OMVs‐associated HapA did not disrupt pre‐formed HCT8 spheroids (Figure 6D).

To further explore whether OMV‐associated HapA affects the tight and adherens junction proteins of the spheroids, we exposed spheroids derived from HCT8 cells to *V. cholerae* OMVs isolated from the A1552 wild type (WT) or the Δ*hapA* mutant, followed by Western blot analysis. We observed a decrease in the expression levels of tight and adherens junction proteins in spheroids subjected to the spheroid prevention experiment (Figure 6E). Additionally, data from the spheroid disruption assay showed that OMV‐associated HapA preferentially targeted the tight junction protein claudin over occludin (Figure 6E). Interestingly, under both the prevention and disruption conditions, the adherens junction protein β‐catenin was identified as a substrate for OMV‐associated HapA (Figure 6E). Taken together, these findings imply that OMV‐associated HapA selectively targets the tight and adherens junction proteins claudin and β‐catenin, rather than occludin, shedding light on the intricate dynamics of bacterial OMV interaction with host cells in a three‐dimensional setting.

**FIGURE 7:**
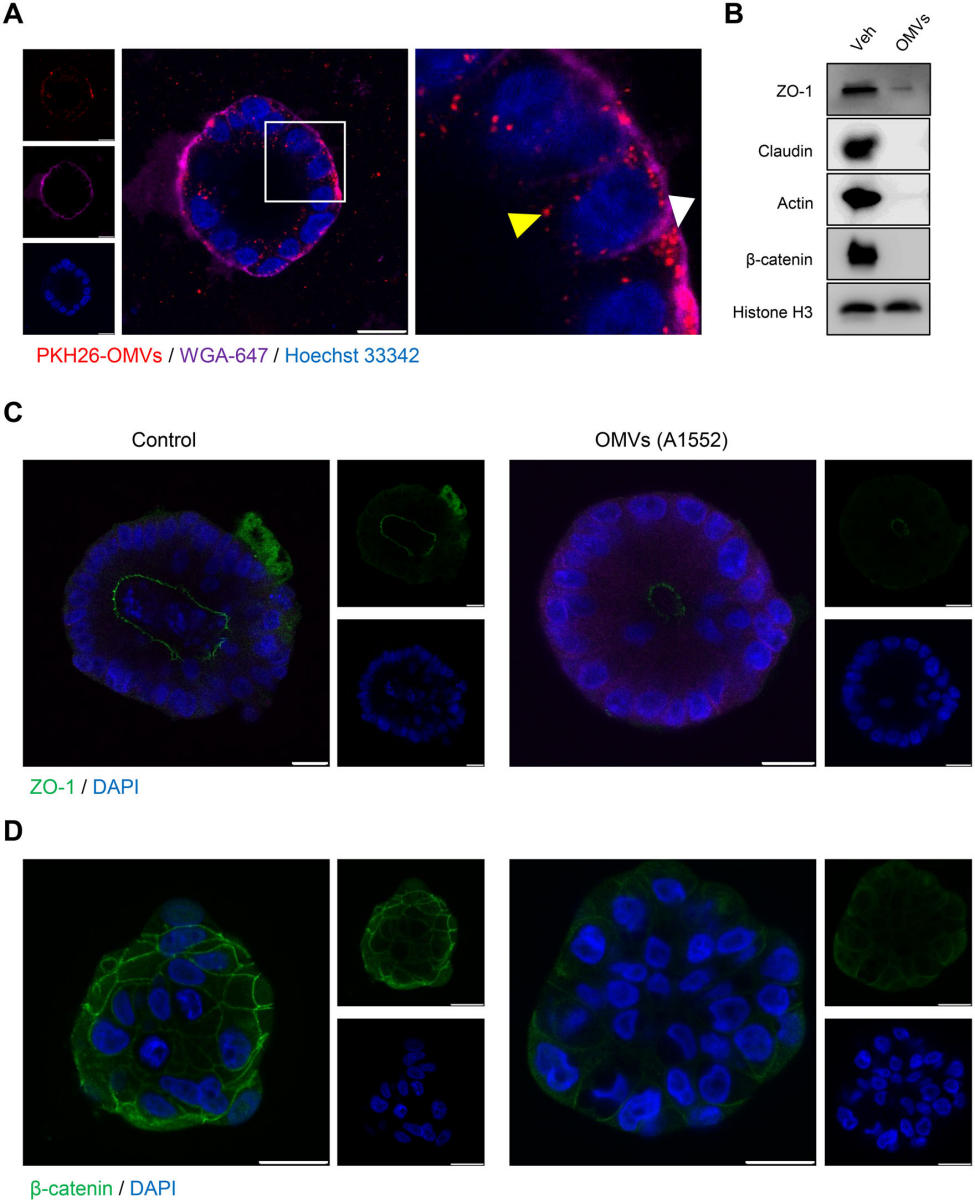
*V. cholerae* OMV‐associated HapA degrades tight‐ and adherens‐junction proteins in intestinal organoids. (A) Intestinal organoids were exposed to PKH26‐labelled OMVs (50 µg/mL, 4 h), followed by confocal microscopy. PKH26‐OMVs accumulated on the cell membrane of organoids (white arrowhead), with some localised close to the lumen (yellow arrowhead). The cell was stained with Alexa‐labelled WGA (WGA‐647, purple), and nuclei were counterstained with Hoechst 33342 (blue). Scale bars = 20 µm. (B) Western blot analysis of intestinal organoids exposed to *V. cholerae* OMVs (50 µg/mL, 18 h) isolated from A1552 WT strain, showing cleavage of tight‐ and adherens‐junction proteins. Histone H3 was used as a loading control. (C and D) Intestinal organoids were exposed to *V. cholerae* OMVs (50 µg/mL) isolated from A1552 WT for 18 h, followed by fixation and staining for tight junction proteins, ZO‐1 (green), and the adherens‐junction protein, β‐catenin (green). Organoids were counterstained with the nuclear marker DAPI (blue) and visualised by confocal microscopy. OMV‐associated HapA caused a decrease in staining of the tight‐junction protein ZO‐1 (Panel C) and the adherens‐junction protein β‐catenin (Panel D).

In subsequent experiments, we investigated the uptake of *V. cholerae* OMVs by intestinal organoids, exposing them to 20 µg/mL of OMVs for 6 h. Confocal microscopy analysis revealed that the majority of the PKH26‐labelled OMVs were localised either on the cell membrane or within the intracellular compartment of the organoids (Figure 7A). Interestingly, we noticed a fraction of PKH26‐OMVs near the apical part of the organoid (Figure 7A). To determine whether the OMV‐associated HapA affects the tight and adherens junction proteins of the organoids, we exposed the organoids to 50 µg/mL of OMVs for 18 h and performed Western blot analysis to detect these proteins. The results suggested that a significant portion of these junction proteins served as substrates for the OMV‐associated HapA (Figure 7B). Immunostaining further validated the reduction in the tight junction protein ZO‐1 and the adherens junction protein β‐catenin (Figures 7C,D and S7). Interestingly, in the control organoids, immunostaining for the tight junction protein ZO‐1 was localised to the apical region of the organoid (basal‐out and apical‐in organoid). Conversely, immunostaining for the junctional protein β‐catenin was predominantly observed at the cell membrane of the 3D organoid structure (Figure 7D). These observations demonstrate that OMV‐associated HapA retains its biological activity in a more physiologically relevant setting, specifically targeting and cleaving tight and adherens junction proteins.

## 4 Discussion

Our study provides profound insights into the pathogenesis of *V. cholerae*, with a particular emphasis on the multifaceted role of *V. cholerae* OMV‐associated HapA in compromising epithelial cell junctions—a critical aspect of *V. cholerae* pathogenesis. We demonstrated that OMV‐associated HapA plays a key role in detaching intestinal epithelial cells and disrupting the integrity of tight and adherens junctions, underscoring its importance in the pathogenesis of cholera. The initial step in our study involved characterising OMVs isolated from the wild‐type *V. cholerae* strain A1552 and its isogenic Δ*hapA* mutant. Transmission electron microscopy (TEM), immunoblot analysis and the azocasein assay were employed to visualise and quantify the presence and activity of HapA within OMVs. These analyses confirmed the association of HapA with OMVs and provided essential insights into its enhanced biological activity. Both TEM and nanoparticle tracking analysis (NTA) revealed the presence of heterogeneous OMV populations, with TEM detecting vesicles in the 20–200 nm range and NTA identifying two predominant peaks at approximately 105 and 175 nm in OMVs from the wild‐type strain (Figure 1A). Similarly, OMVs from the Δ*hapA* mutant exhibited size heterogeneity, with NTA detecting three major peaks at 55, 125 and 255 nm. It is important to note that while both NTA and TEM revealed size heterogeneity, the techniques differ in sensitivity and resolution. In particular, NTA may under‐represent the abundance of smaller OMVs (<50 nm), as its detection threshold is limited by Brownian motion and light scattering sensitivity (Comfort et al. 2021; Kowkabany and Bao 2024). In contrast, TEM allows direct visualisation of smaller vesicles, including those that may be missed by NTA. This discrepancy likely explains why smaller OMV populations were more readily detected by TEM and in fractions enriched via OptiPrep density gradient centrifugation, but were less prominent in NTA profiles. Consistent with the presence of multiple vesicle populations, immunoblot analysis of gradient‐separated fractions revealed that the larger OMVs were more abundantly associated with HapA (Figure S1C), suggesting a size‐dependent distribution of this protein.

Notably, only the 35 kDa processed form of HapA was released from *V. cholerae* in association with OMVs, and this form exhibited significantly more pronounced effects on host cells compared to the purified 35 kDa HapA protein, despite both being the same molecular size. Previous studies on the *V. cholerae* non‐O1, non‐O139 strain PL‐21 demonstrated distinct activities for the two forms of HapA: the 35 kDa form induced haemorrhagic fluid response, inflammation and necrosis in rabbit ileum, whereas the 45 kDa form increased intestinal short‐circuit current and caused cell distention in HeLa cells (Ghosh et al. 2006). In contrast, OMVs from *E. coli* expressing HapA were associated with the 45 kDa form, suggesting that HapA processing is species‐ and strain‐dependent, possibly influenced by additional cellular factors. Supporting this idea, earlier work with the *V. cholerae* strain C6709 indicated that HapA was released in association with OMVs as a 45 kDa protein (Mondal et al. 2016). These findings suggest that the processed or mature form of HapA present in culture supernatants may vary by strain. Our results demonstrate that OMV‐associated HapA triggers a faster cellular detachment response compared to the purified protein, implying that its encapsulation within OMVs enhances its activity. This enhancement could be attributed to increased stability, more efficient cellular uptake or more targeted delivery to specific sites, further reinforcing the critical role of OMVs in *V. cholerae* virulence.

The integrity of the epithelial barriers, maintained by TJs and AJs, is crucial for protecting against pathogenic invasion. Our findings demonstrate that OMV‐associated HapA specifically disrupts this barrier by reducing the levels of key junctional proteins, including claudin, ZO‐1 and β‐catenin, as confirmed by confocal microscopy and Western blot analysis. This targeted disruption of junctional proteins by HapA highlights its proposed role in bacterial virulence by weakening epithelial barriers, potentially facilitating bacterial invasion and colonisation. Moreover, our studies revealed the rapid internalisation of *V. cholerae* OMVs by both polarised and non‐polarised epithelial cells, involving caveolin‐, clathrin‐ and macropinocytosis‐dependent pathways, similar to OMVs from other Gram‐negative bacterial species (Magana et al. 2023; O'Donoghue and Krachler 2016). This suggests a sophisticated mechanism for delivering bacterial virulence factors into host cells, expanding our understanding of *V. cholerae* infection strategies.

Our observation of HapA's specific targeting of tight and adherens junctions provides a new perspective on cholera pathogenesis, proposing that the disruption of these junctions may contribute to the characteristically severe diarrhoea of cholera. HapA's role extends beyond this, as it has been shown to enhance the activity of other toxins, degrade the protective mucus barrier, and promote bacterial dissemination throughout the gastrointestinal tract (Finkelstein et al. 1983; Sikora et al. 2011; Tsou and Zhu 2010). These activities collectively facilitate the access of vibrio's or their toxins to the microvilli beneath the mucus barrier and support the spread of bacteria within the host (Finkelstein et al. 1992; Jude et al. 2009; Mel et al. 2000; Silva et al. 2003).

HapA is also known to play significant roles in the environmental adaptation of *V. cholerae*. It has been implicated in interactions with aquatic hosts, such as fish, where it is highly prevalent in strains isolated from fish intestines (Xu et al. 2019). Additionally, quorum sensing regulates HapA's proteolytic activity to control multicellular aggregation and void formation, essential for community lifestyle commitment, highlighting its dual role in both environmental adaptation and pathogenesis (Caigoy et al. 2022; Jemielita et al. 2021). Furthermore, *V. cholerae*'s production of HapA can be triggered by external cues, such as autoinducer‐2 (AI‐2) from chironomid egg mass bacteria, enhancing HapA activity and contributing to egg mass degradation (Sela et al. 2021). These insights highlight the complexity of HapA's roles in both environmental adaptation and pathogenesis.

Our use of intestinal organoids also represents a significant advancement in *V. cholerae* research, using intestinal organoids to investigate HapA‐host interactions. Organoids offer a more physiologically relevant and ethically preferable alternative to traditional animal models, closely replicating the intricate structure and function of human tissues. In our investigation, the use of organoids facilitated a detailed examination of the effects of HapA on epithelial cell junctions and barrier function, offering insights that might be less discernible in conventional in vitro models.

Our findings, coupled with the demonstrated utility of organoids in assessing cholera toxin inhibitors, underscore the value of this innovative approach in studying *V. cholerae* pathogenesis (Haksar et al. 2019; Zomer‐van Ommen et al. 2016). While the concentrations of OMVs used in our experiments (20–50 µg/mL, based on protein content) may appear high, defining physiologically relevant OMV levels during *V. cholerae* infection is currently limited by the lack of quantitative in vivo data. For confocal imaging, we used 20 µg/mL to minimise morphological changes, whereas 50 µg/mL was applied in organoid assays to ensure sufficient biological response. These concentrations are consistent with previous functional OMV studies. For instance, we previously used 25–50 µg/mL of *V. cholerae* OMVs to activate cGAS–STING signalling in vivo without adverse effects (Erttmann et al. 2022). Similarly, other studies have used comparable or higher concentrations, for example, 20–40 and 50 µg/mL in epithelial co‐culture assays (Chatterjee and Chaudhuri 2013; Kesty et al. 2004) and 5–25 µg/mL for investigating the interaction of *Legionella pneumophila* OMVs with mammalian host cells (Jager et al. 2015). These precedents support the experimental relevance of the OMV concentrations used in our study. Recent studies using human enteroids have elucidated key mechanisms of *V. cholerae* infection, including how cholera toxin binds and intoxicates host cells through glycosphingolipids and glycoproteins, further demonstrating the relevance of these models in infection research (Singla et al. 2023).

In conclusion, our research significantly advances the understanding of *V. cholerae* pathogenesis, particularly by elucidating how OMV‐associated HapA disrupts epithelial cell junctions. These findings contribute to a broader understanding of bacterial pathogenesis and provide valuable insights that may inform the development of new therapeutic strategies to combat cholera, which remains a global health challenge.

## Author Contributions

P.B. performed isolation of OMVs, confocal microscopy, immunoblot analysis, flow cytometry, cell toxicity, 3D spheroids and organoids culture; D.I. performed isolation of OMVs, immunoblots, nanoparticle tracking analysis, 3D spheroids and cell toxicity; E.T. performed isolation of OMVs, immunoblot analysis, cell toxicity, 3D spheroids and organoids culture; A.N. performed confocal microscopy and analysed the data. M.R. performed cryo‐XPS analyses. P.B. and A.N. wrote the initial version of the manuscript. All authors read and commented on the manuscript. B.E.U. and S.N.W. obtained the funding. A. N., B.E.U. and S.N.W. supervised the research and finalised the manuscript.

## Conflicts of Interest

The authors declare no competing interests.

## Supporting information



Supporting Information

Supporting Information

Supporting Information

Supporting Information

Supporting Information

Supporting Information

Supporting Information

Supporting Information

Supporting Information

## Data Availability

The data that support the findings of this study are available from the corresponding author upon reasonable request.

## References

[jev270092-bib-0001] Benitez, J. A. , and A. J. Silva . 2016. “Vibrio Cholerae Hemagglutinin(HA)/Protease: An Extracellular Metalloprotease With Multiple Pathogenic Activities.” Toxicon 115: 55–62. 26952544 10.1016/j.toxicon.2016.03.003 PMC4828278

[jev270092-bib-0002] Bermudez‐Brito, M. , J. Plaza‐Diaz , L. Fontana , S. Munoz‐Quezada , and A. Gil . 2013. “In Vitro Cell and Tissue Models for Studying Host‐microbe Interactions: A Review.” British Journal of Nutrition 109, no. 2: S27–34. 23360878 10.1017/S0007114512004023

[jev270092-bib-0003] Bitar, A. , K. M. Aung , S. N. Wai , and M. L. Hammarstrom . 2019. “Vibrio Cholerae Derived Outer Membrane Vesicles Modulate the Inflammatory Response of Human Intestinal Epithelial Cells by Inducing MicroRNA‐146a.” Scientific Reports 9: 7212. 31076615 10.1038/s41598-019-43691-9 PMC6510749

[jev270092-bib-0004] Booth, B. A. , M. Boesman‐Finkelstein , and R. A. Finkelstein . 1983. “Vibrio Cholerae Soluble Hemagglutinin/Protease Is a Metalloenzyme.” Infection and Immunity 42: 639–644. 6417020 10.1128/iai.42.2.639-644.1983 PMC264477

[jev270092-bib-0005] Booth, B. A. , M. Boesman‐Finkelstein , and R. A. Finkelstein . 1984. “Vibrio Cholerae Hemagglutinin/Protease Nicks Cholera Enterotoxin.” Infection and Immunity 45: 558–560. 6432694 10.1128/iai.45.3.558-560.1984 PMC263329

[jev270092-bib-0006] Caigoy, J. C. , T. Shimamoto , A. K. Mukhopadhyay , S. Shinoda , and T. Shimamoto . 2022. “Sequence Polymorphisms in Vibrio Cholerae HapR Affect Biofilm Formation Under Aerobic and Anaerobic Conditions.” Applied and Environmental Microbiology 88: e0104422. 35969071 10.1128/aem.01044-22 PMC9469714

[jev270092-bib-0007] Chatterjee, D. , and K. Chaudhuri . 2013. “Vibrio Cholerae O395 Outer Membrane Vesicles Modulate Intestinal Epithelial Cells in a NOD1 Protein‐Dependent Manner and Induce Dendritic Cell‐Mediated Th2/Th17 Cell Responses.” Journal of Biological Chemistry 288: 4299–4309. 23275338 10.1074/jbc.M112.408302 PMC3567681

[jev270092-bib-0008] Chatterjee, S. , K. Ghosh , A. Raychoudhuri , et al. 2009. “Incidence, Virulence Factors, and Clonality Among Clinical Strains of Non‐O1, Non‐O139 Vibrio Cholerae Isolates From Hospitalized Diarrheal Patients in Kolkata, India.” Journal of Clinical Microbiology 47: 1087–1095. 19158257 10.1128/JCM.02026-08 PMC2668327

[jev270092-bib-0009] Comfort, N. , K. Cai , T. R. Bloomquist , M. D. Strait , A. W. Ferrante Jr. , and A. A. Baccarelli . 2021. “Nanoparticle Tracking Analysis for the Quantification and Size Determination of Extracellular Vesicles.” Journal of Visualized Experiments: JoVE 169: 1–33. 10.3791/62447 PMC8243380 33843938

[jev270092-bib-0010] Crowther, R. S. , N. W. Roomi , R. E. Fahim , and J. F. Forstner . 1987. “Vibrio Cholerae Metalloproteinase Degrades Intestinal Mucin and Facilitates Enterotoxin‐Induced Secretion From Rat Intestine.” Biochimica Et Biophysica Acta 924: 393–402. 3297167 10.1016/0304-4165(87)90153-x

[jev270092-bib-0011] Dutta, D. , and H. Clevers . 2017. “Organoid Culture Systems to Study Host‐Pathogen Interactions.” Current Opinion in Immunology 48: 15–22. 28756233 10.1016/j.coi.2017.07.012 PMC7126332

[jev270092-bib-0012] Elluri, S. , C. Enow , S. Vdovikova , et al. 2014. “Outer Membrane Vesicles Mediate Transport of Biologically Active Vibrio Cholerae Cytolysin (VCC) From *V. cholerae* Strains.” PLoS ONE 9: e106731. 25187967 10.1371/journal.pone.0106731 PMC4154730

[jev270092-bib-0013] Erttmann, S. F. , P. Swacha , K. M. Aung , et al. 2022. “The Gut Microbiota Prime Systemic Antiviral Immunity via the cGAS‐STING‐IFN‐I Axis.” Immunity 55: 847–861. e810. 35545033 10.1016/j.immuni.2022.04.006

[jev270092-bib-0014] Farquhar, M. G. , and G. E. Palade . 1963. “Junctional Complexes in Various Epithelia.” Journal of Cell Biology 17: 375–412. 13944428 10.1083/jcb.17.2.375 PMC2106201

[jev270092-bib-0015] Finkelstein, R. A. , M. Boesman‐Finkelstein , Y. Chang , and C. C. Hase . 1992. “Vibrio Cholerae Hemagglutinin/Protease, Colonial Variation, Virulence, and Detachment.” Infection and Immunity 60: 472–478. 1730478 10.1128/iai.60.2.472-478.1992 PMC257651

[jev270092-bib-0016] Finkelstein, R. A. , M. Boesman‐Finkelstein , and P. Holt . 1983. “Vibrio Cholerae Hemagglutinin/Lectin/Protease Hydrolyzes Fibronectin and Ovomucin: F.M. Burnet Revisited.” Proceedings of the National Academy of Sciences of the United States of America 80: 1092–1095. 6341990 10.1073/pnas.80.4.1092 PMC393534

[jev270092-bib-0017] Finkelstein, R. A. , and L. F. Hanne . 1982. “Purification and Characterization of the Soluble Hemagglutinin (Cholera Lectin)(Produced by Vibrio cholerae).” Infection and Immunity 36: 1199–1208. 7047394 10.1128/iai.36.3.1199-1208.1982 PMC551457

[jev270092-bib-0018] Fujimoto, L. M. , R. Roth , J. E. Heuser , and S. L. Schmid . 2000. “Actin Assembly Plays a Variable, but Not Obligatory Role in Receptor‐Mediated Endocytosis in Mammalian Cells.” Traffic (Copenhagen, Denmark) 1: 161–171. 11208096 10.1034/j.1600-0854.2000.010208.x

[jev270092-bib-0019] Furuse, M. , M. Hata , K. Furuse , et al. 2002. “Claudin‐Based Tight Junctions Are Crucial for the Mammalian Epidermal Barrier: A Lesson From Claudin‐1‐Deficient Mice.” Journal of Cell Biology 156: 1099–1111. 11889141 10.1083/jcb.200110122 PMC2173463

[jev270092-bib-0020] Gartler, S. M. 1968. “Apparent Hela Cell Contamination of Human Heteroploid Cell Lines.” Nature 217: 750–751. 5641128 10.1038/217750a0

[jev270092-bib-0021] Ghosh, A. , D. R. Saha , K. M. Hoque , et al. 2006. “Enterotoxigenicity of Mature 45‐Kilodalton and Processed 35‐Kilodalton Forms of Hemagglutinin Protease Purified From a Cholera Toxin Gene‐Negative Vibrio Cholerae Non‐O1, Non‐O139 Strain.” Infection and Immunity 74: 2937–2946. 16622232 10.1128/IAI.74.5.2937-2946.2006 PMC1459690

[jev270092-bib-0022] Guttman, J. A. , and B. B. Finlay . 2009. “Tight Junctions as Targets of Infectious Agents.” Biochimica Et Biophysica Acta 1788: 832–841. 19059200 10.1016/j.bbamem.2008.10.028

[jev270092-bib-0023] Haksar, D. , E. de Poel , L. Q. van Ufford , et al. 2019. “Strong Inhibition of Cholera Toxin B Subunit by Affordable, Polymer‐Based Multivalent Inhibitors.” Bioconjugate Chemistry 30: 785–792. 30629410 10.1021/acs.bioconjchem.8b00902 PMC6429436

[jev270092-bib-0024] Ikenouchi, J. , M. Matsuda , M. Furuse , and S. Tsukita . 2003. “Regulation of Tight Junctions During the Epithelium‐Mesenchyme Transition: Direct Repression of the Gene Expression of Claudins/Occludin by Snail.” Journal of Cell Science 116: 1959–1967. 12668723 10.1242/jcs.00389

[jev270092-bib-0025] Jager, J. , S. Keese , M. Roessle , M. Steinert , and A. B. Schromm . 2015. “Fusion of Legionella Pneumophila Outer Membrane Vesicles With Eukaryotic Membrane Systems Is a Mechanism to Deliver Pathogen Factors to Host Cell Membranes.” Cellular Microbiology 17: 607–620. 25363599 10.1111/cmi.12392

[jev270092-bib-0026] Jemielita, M. , A. A. Mashruwala , J. S. Valastyan , N. S. Wingreen , and B. L. Bassler . 2021. “Secreted Proteases Control the Timing of Aggregative Community Formation in Vibrio Cholerae.” mBio 12: e0151821. 34809464 10.1128/mBio.01518-21 PMC8609355

[jev270092-bib-0027] Jude, B. A. , R. M. Martinez , K. Skorupski , and R. K. Taylor . 2009. “Levels of the Secreted Vibrio Cholerae Attachment Factor GbpA Are Modulated by Quorum‐Sensing‐Induced Proteolysis.” Journal of Bacteriology 191: 6911–6917. 19734310 10.1128/JB.00747-09 PMC2772460

[jev270092-bib-0028] Kapalczynska, M. , T. Kolenda , W. Przybyla , et al. 2018. “2D and 3D Cell Cultures—A Comparison of Different Types of Cancer Cell Cultures.” Archives of Medical Science 14: 910–919. 30002710 10.5114/aoms.2016.63743 PMC6040128

[jev270092-bib-0029] Kaper, J. B. , J. G. Morris Jr. , and M. M. Levine . 1995. “Cholera.” Clinical Microbiology Reviews 8: 48–86. 7704895 10.1128/cmr.8.1.48 PMC172849

[jev270092-bib-0030] Kathuria, R. , and K. Chattopadhyay . 2018. “Vibrio Cholerae Cytolysin: Multiple Facets of the Membrane Interaction Mechanism of a Beta‐Barrel Pore‐Forming Toxin.” IUBMB Life 70: 260–266. 29469977 10.1002/iub.1725

[jev270092-bib-0031] Kesty, N. C. , K. M. Mason , M. Reedy , S. E. Miller , and M. J. Kuehn . 2004. “Enterotoxigenic *Escherichia coli* Vesicles Target Toxin Delivery Into Mammalian Cells.” Embo Journal 23: 4538–4549. 15549136 10.1038/sj.emboj.7600471 PMC533055

[jev270092-bib-0032] Konig, J. , J. Wells , P. D. Cani , et al. 2016. “Human Intestinal Barrier Function in Health and Disease.” Clinical and Translational Gastroenterology 7: e196. 27763627 10.1038/ctg.2016.54 PMC5288588

[jev270092-bib-0033] Kowkabany, G. , and Y. Bao . 2024. “Nanoparticle Tracking Analysis: An Effective Tool to Characterize Extracellular Vesicles.” Molecules (Basel, Switzerland) 29: 4672. 39407601 10.3390/molecules29194672 PMC11477862

[jev270092-bib-0034] Krebs, S. J. , and R. K. Taylor . 2011. “Protection and Attachment of Vibrio Cholerae Mediated by the Toxin‐Coregulated Pilus in the Infant Mouse Model.” Journal of Bacteriology 193: 5260–5270. 21804008 10.1128/JB.00378-11 PMC3187450

[jev270092-bib-0035] Magana, G. , C. Harvey , C. C. Taggart , and A. M. Rodgers . 2023. “Bacterial Outer Membrane Vesicles: Role in Pathogenesis and Host‐Cell Interactions.” Antibiotics (Basel) 13: 32. 38247591 10.3390/antibiotics13010032 PMC10812699

[jev270092-bib-0036] Mel, S. F. , K. J. Fullner , S. Wimer‐Mackin , W. I. Lencer , and J. J. Mekalanos . 2000. “Association of Protease Activity in *Vibrio cholerae* Vaccine Strains With Decreases in Transcellular Epithelial Resistance of Polarized T84 Intestinal Epithelial Cells.” Infection and Immunity 68: 6487–6492. 11035765 10.1128/iai.68.11.6487-6492.2000 PMC97739

[jev270092-bib-0037] Miyoshi, S. 2013. “Extracellular Proteolytic Enzymes Produced by Human Pathogenic Vibrio Species.” Frontiers in Microbiology 4: 339. 24302921 10.3389/fmicb.2013.00339 PMC3831164

[jev270092-bib-0038] Mondal, A. , R. Tapader , N. S. Chatterjee , et al. 2016. “Cytotoxic and Inflammatory Responses Induced by Outer Membrane Vesicle‐Associated Biologically Active Proteases From *Vibrio cholerae* .” Infection and Immunity 84: 1478–1490. 26930702 10.1128/IAI.01365-15 PMC4862697

[jev270092-bib-0068] Montero, D. A. , R. M. Vidal , J. Velasco , et al. 2023. “ *Vibrio cholerae*, classification, pathogenesis, immune response, and trends in vaccine development.” Frontiersd in Medicine 10: 1–24. 10.3389/fmed.2023.1155751 PMC10196187 37215733

[jev270092-bib-0039] Nadeem, A. , A. Alam , E. Toh , et al. 2021. “Phosphatidic Acid‐Mediated Binding and Mammalian Cell Internalization of the *Vibrio cholerae* Cytotoxin MakA.” PLOS Pathogens 17: e1009414. 33735319 10.1371/journal.ppat.1009414 PMC8009392

[jev270092-bib-0040] Nadeem, A. , J. Oscarsson , and S. N. Wai . 2020. Delivery of Virulence Factors by Bacterial Membrane Vesicles to Mammalian Host Cells. Springer Nature.

[jev270092-bib-0041] Nagamune, K. , K. Yamamoto , A. Naka , J. Matsuyama , T. Miwatani , and T. Honda . 1996. “In Vitro Proteolytic Processing and Activation of the Recombinant Precursor of El Tor Cytolysin/Hemolysin (pro‐HlyA) of *Vibrio cholerae* by Soluble Hemagglutinin/Protease of *V. cholerae*, Trypsin, and Other Proteases.” Infection and Immunity 64: 4655–4658. 8890221 10.1128/iai.64.11.4655-4658.1996 PMC174427

[jev270092-bib-0042] O'Donoghue, E. J. , and A. M. Krachler . 2016. “Mechanisms of Outer Membrane Vesicle Entry Into Host Cells.” Cellular Microbiology 18: 1508–1517. 27529760 10.1111/cmi.12655 PMC5091637

[jev270092-bib-0043] O'Donoghue, E. J. , N. Sirisaengtaksin , D. F. Browning , et al. 2017. “Lipopolysaccharide Structure Impacts the Entry Kinetics of Bacterial Outer Membrane Vesicles Into Host Cells.” PLOS Pathogens 13: e1006760. 29186191 10.1371/journal.ppat.1006760 PMC5724897

[jev270092-bib-0044] Ramstedt, M. , R. Nakao , S. N. Wai , B. E. Uhlin , and J. F. Boily . 2011. “Monitoring Surface Chemical Changes in the Bacterial Cell Wall: Multivariate Analysis of Cryo‐X‐Ray Photoelectron Spectroscopy Data.” Journal of Biological Chemistry 286: 12389–12396. 21330374 10.1074/jbc.M110.209536 PMC3069442

[jev270092-bib-0045] Ramstedt, M. , and A. Shchukarev . 2016. “Analysis of Bacterial Cell Surface Chemical Composition Using Cryogenic X‐Ray Photoelectron Spectroscopy.” Methods in Molecular Biology 1440: 215–223. 27311675 10.1007/978-1-4939-3676-2_16

[jev270092-bib-0046] Rompikuntal, P. K. , S. Vdovikova , M. Duperthuy , et al. 2015. “Outer Membrane Vesicle‐Mediated Export of Processed PrtV Protease From *Vibrio cholerae* .” PLoS ONE 10: e0134098. 26222047 10.1371/journal.pone.0134098 PMC4519245

[jev270092-bib-0047] Schindelin, J. , I. Arganda‐Carreras , E. Frise , et al. 2012. “Fiji: An Open‐Source Platform for Biological‐Image Analysis.” Nature Methods 9: 676–682. 22743772 10.1038/nmeth.2019 PMC3855844

[jev270092-bib-0048] Sela, R. , B. K. Hammer , and M. Halpern . 2021. “Quorum‐Sensing Signaling by Chironomid Egg Masses' Microbiota, Affects Haemagglutinin/Protease (HAP) Production by *Vibrio cholerae* .” Molecular Ecology 30: 1736–1746. 33001525 10.1111/mec.15662

[jev270092-bib-0049] Shchukarev, A. , E. Backman , S. Watts , S. Salentinig , C. F. Urban , and M. Ramstedt . 2021. “Applying Cryo‐X‐Ray Photoelectron Spectroscopy to Study the Surface Chemical Composition of Fungi and Viruses.” Frontiers in Chemistry 9: 666853. 34124001 10.3389/fchem.2021.666853 PMC8194281

[jev270092-bib-0050] Sikora, A. E. , R. A. Zielke , D. A. Lawrence , P. C. Andrews , and M. Sandkvist . 2011. “Proteomic Analysis of the *Vibrio cholerae* Type II Secretome Reveals New Proteins, Including Three Related Serine Proteases.” Journal of Biological Chemistry 286: 16555–16566. 21385872 10.1074/jbc.M110.211078 PMC3089498

[jev270092-bib-0051] Silva, A. J. , K. Pham , and J. A. Benitez . 2003. “Haemagglutinin/Protease Expression and Mucin Gel Penetration in El Tor Biotype *Vibrio cholerae* .” Microbiology (NY Reading) 149: 1883–1891. 10.1099/mic.0.26086-0 12855739

[jev270092-bib-0052] Singla, A. , A. Boucher , K. L. Wallom , et al. 2023. “Cholera Intoxication of Human Enteroids Reveals Interplay Between Decoy and Functional Glycoconjugate Ligands.” Glycobiology 33: 801–816. 37622990 10.1093/glycob/cwad069 PMC10629719

[jev270092-bib-0053] Stevenson, B. R. , J. D. Siliciano , M. S. Mooseker , and D. A. Goodenough . 1986. “Identification of ZO‐1: A High Molecular Weight Polypeptide Associated With the Tight Junction (Zonula Occludens) in a Variety of Epithelia.” Journal of Cell Biology 103: 755–766. 3528172 10.1083/jcb.103.3.755 PMC2114282

[jev270092-bib-0054] Stewart‐Tull, D. E. , R. A. Ollar , and T. S. Scobie . 1986. “Studies on the *Vibrio cholerae* Mucinase Complex. I. Enzymic Activities Associated With the Complex.” Journal of Medical Microbiology 22: 325–333. 3025445 10.1099/00222615-22-4-325

[jev270092-bib-0055] Trucksis, M. , J. E. Galen , J. Michalski , A. Fasano , and J. B. Kaper . 1993. “Accessory Cholera Enterotoxin (Ace), the Third Toxin of a *Vibrio cholerae* Virulence Cassette.” Proceedings of the National Academy of Sciences of the United States of America 90: 5267–5271. 8389476 10.1073/pnas.90.11.5267 PMC46697

[jev270092-bib-0056] Tsou, A. M. , and J. Zhu . 2010. “Quorum Sensing Negatively Regulates Hemolysin Transcriptionally and Posttranslationally in *Vibrio cholerae* .” Infection and Immunity 78: 461–467. 19858311 10.1128/IAI.00590-09 PMC2798175

[jev270092-bib-0057] Vaitkevicius, K. , B. Lindmark , G. Ou , et al. 2006. “A *Vibrio cholerae* Protease Needed for Killing of Caenorhabditis Elegans Has a Role in Protection From Natural Predator Grazing.” Proceedings of the National Academy of Sciences of the United States of America 103: 9280–9285. 16754867 10.1073/pnas.0601754103 PMC1482601

[jev270092-bib-0058] Van der Henst, C. , A. S. Vanhove , N. C. Drebes Dorr , et al. 2018. “Molecular Insights Into *Vibrio cholerae's* Intra‐Amoebal Host‐Pathogen Interactions.” Nature Communications 9: 3460. 10.1038/s41467-018-05976-x PMC6110790 30150745

[jev270092-bib-0059] Wai, S. N. , B. Lindmark , T. Soderblom , et al. 2003. “Vesicle‐Mediated Export and Assembly of Pore‐Forming Oligomers of the Enterobacterial ClyA Cytotoxin.” Cell 115: 25–35. 14532000 10.1016/s0092-8674(03)00754-2

[jev270092-bib-0060] Weil, A. A. , R. L. Becker , and J. B. Harris . 2019. “ *Vibrio cholerae* at the Intersection of Immunity and the Microbiome.” mSphere 4: e00597–519. 10.1128/mSphere.00597-19 PMC6881719 31776240

[jev270092-bib-0061] Wu, Z. , D. Milton , P. Nybom , A. Sjo , and K. E. Magnusson . 1996. “ *Vibrio cholerae* Hemagglutinin/Protease (HA/Protease) Causes Morphological Changes in Cultured Epithelial Cells and Perturbs Their Paracellular Barrier Function.” Microbial Pathogenesis 21: 111–123. 8844654 10.1006/mpat.1996.0047

[jev270092-bib-0062] Wu, Z. , P. Nybom , and K. E. Magnusson . 2000. “Distinct Effects of *Vibrio cholerae* Haemagglutinin/Protease on the Structure and Localization of the Tight Junction‐Associated Proteins Occludin and ZO‐1.” Cellular Microbiology 2: 11–17. 11207559 10.1046/j.1462-5822.2000.00025.x

[jev270092-bib-0063] Xu, M. , H. Fu , D. Chen , et al. 2019. “Simple Visualized Detection Method of Virulence‐Associated Genes of *Vibrio cholerae* by Loop‐Mediated Isothermal Amplification.” Frontiers in Microbiology 10: 2899. 31921074 10.3389/fmicb.2019.02899 PMC6932958

[jev270092-bib-0064] Yarar, D. , C. M. Waterman‐Storer , and S. L. Schmid . 2005. “A Dynamic Actin Cytoskeleton Functions at Multiple Stages of Clathrin‐Mediated Endocytosis.” Molecular Biology of the Cell 16: 964–975. 15601897 10.1091/mbc.E04-09-0774 PMC545926

[jev270092-bib-0065] Zhu, J. , and J. J. Mekalanos . 2003. “Quorum Sensing‐Dependent Biofilms Enhance Colonization in *Vibrio cholerae* .” Developmental Cell 5: 647–656. 14536065 10.1016/s1534-5807(03)00295-8

[jev270092-bib-0066] Zlatkov, N. , A. Nadeem , B. E. Uhlin , and S. N. Wai . 2021. “Eco‐Evolutionary Feedbacks Mediated by Bacterial Membrane Vesicles.” FEMS Microbiology Review 45: fuaa047. 10.1093/femsre/fuaa047 PMC7968517 32926132

[jev270092-bib-0067] Zomer‐van Ommen, D. D. , A. V. Pukin , O. Fu , et al. 2016. “Functional Characterization of Cholera Toxin Inhibitors Using Human Intestinal Organoids.” Journal of Medicinal Chemistry 59: 6968–6972. 27347611 10.1021/acs.jmedchem.6b00770

