# Supplementary material for: Hemagglutinin Protease HapA Associated With Vibrio cholerae Outer Membrane Vesicles (OMVs) Disrupts Tight and Adherens Junctions
Source: J Extracell Vesicles. 2025 May 25;14(5):e70092. doi: 10.1002/jev2.70092 (PMC12104216; doi:10.1002/jev2.70092)
Supplement: Supplementary file 1 — Supporting Information [file JEV2-14-e70092-s002.docx]

**Supplementary Information**

***Vibrio cholerae* OMV-associated HapA causes disruption of tight and adherens junctions in 2D and 3D cell models.**

Palwasha Baryalai^1,2^, David Irenaeus^1^, Eric Toh^1,2^, Madeleine Ramstedt^3^, Bernt Eric Uhlin^1^, Aftab Nadeem^1*^, Sun Nyunt Wai1,2*

1Department of Molecular Biology and Umeå Centre for Microbial Research (UCMR), Umeå University, SE-90187 Umeå, Sweden

2The Laboratory for Molecular Infection Medicine Sweden (MIMS), Umeå University, SE-90187 Umeå, Sweden

^3^Department of Chemistry and Umeå Centre for Microbial Research (UCMR), Umeå University, 901 87 Umeå, Sweden.

* Corresponding authors:

Aftab Nadeem, E-mail: aftab.nadeem@umu.se

Sun Nyunt Wai, E-mail: [sun.nyunt.wai@umu.se](mailto:sun.nyunt.wai@umu.se)


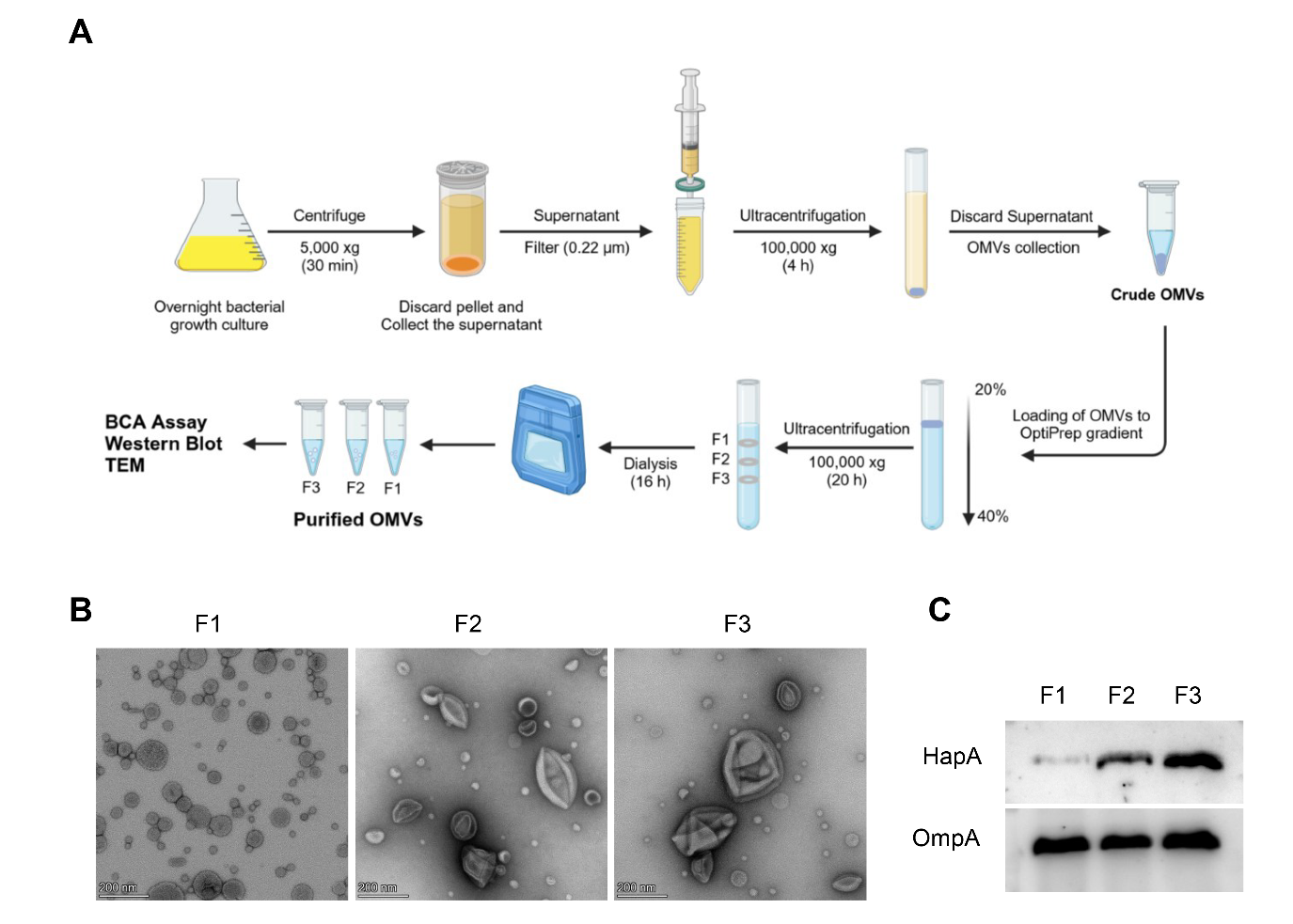


**Figure S1: HapA is enriched in larger OMV fractions.**

**(A)** Schematic overview of the workflow for OMV isolation and separation by OptiPrep™ density gradient centrifugation. OMVs were first isolated from overnight cultures of V. cholerae by sequential centrifugation and filtration, followed by ultracentrifugation. Crude OMVs were then subjected to density gradient centrifugation and fractionation (F1–F3), followed by dialysis. **(B)** Negative staining electron micrographs of OMV fractions F1-F3. Scale bars, 100 nm. **(C)** Immunoblot analysis of OMVs from each gradient fraction using anti-HapA polyclonal antiserum (upper panel) and anti-OmpA polyclonal antiserum (lower panel). OmpA serves as a marker for OMVs and loading control. HapA is predominantly detected in the large vesicle-enriched fractions (F2 and F3), indicating its enrichment in larger OMVs.


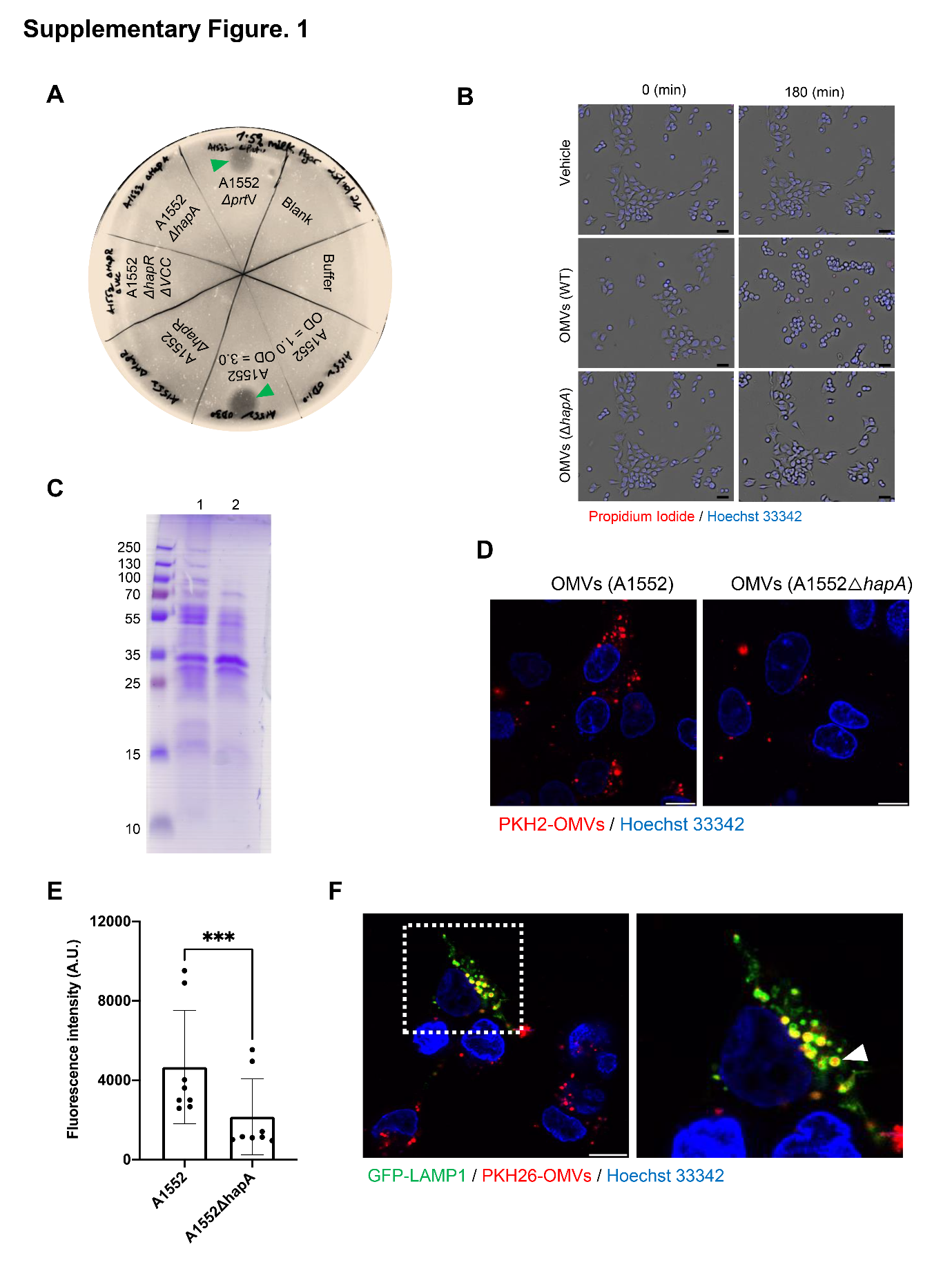


**Figure S2: *V. cholerae* OMVs rapidly accumulate in the endolysosomal compartment of the epithelial cells, and HapA-associated OMVs induce time-dependent rounding of HCT8 cells.**

**(A)** The proteolytic activity of the *V. cholerae* OMVs (10 μg) isolated from different *V. cholerae* strains was tested by spotting them on a milk agar plate. **(B)** HCT8 cells were exposed to OMVs (25 μg/mL) or equal volume PBS as the vehicle control, followed by staining with propidium iodide (red) and Hoechst 33342 (blue). Live imaging was performed using SparkCyto imaging system. OMVs induced a time-dependent rounding and detachment of HCT8 cells. Scale bars, 20 μm. **(C)** SDS-PAGE with Coomassie blue staining, illustrating the protein profiles of OMVs. Lane 1, *E. coli* strain MC1061 harboring HapA expressing plasmid (MC1061/p*hapA*^+^); lane 2, MC1061 carrying the empty plasmid pBAD18 (MC1061/pBAD18). **(D)** Confocal microscopy of HCT8 cells treated for 4 h with PKH26-labeled OMVs (20 μg/mL) from wild-type *V. cholerae* A1552 (WT) or A1552 lacking HapA (Δ*hapA* OMVs). OMVs (red) were visualized along with nuclear counterstaining (Hoechst 33342, blue). **(E)** Flow cytometry comparing the uptake of PKH26-labeled OMVs from A1552 (WT) and Δ*hapA* strains. Data represent replicates (n=8), with bar graphs showing mean ± s.d. Statistical significance was assessed using a non-parametric t-test. **(F)** HCT8 cells transfected with GFP-LAMP1 (green, 24 h) were treated with PKH26-OMVs (20 μg/mL, 4 h, red). Nuclei were stained with Hoechst 33342 (blue). Arrowhead (white) indicates accumulation of OMVs in the lysosomal compartment. Scale bars, 10 μm.


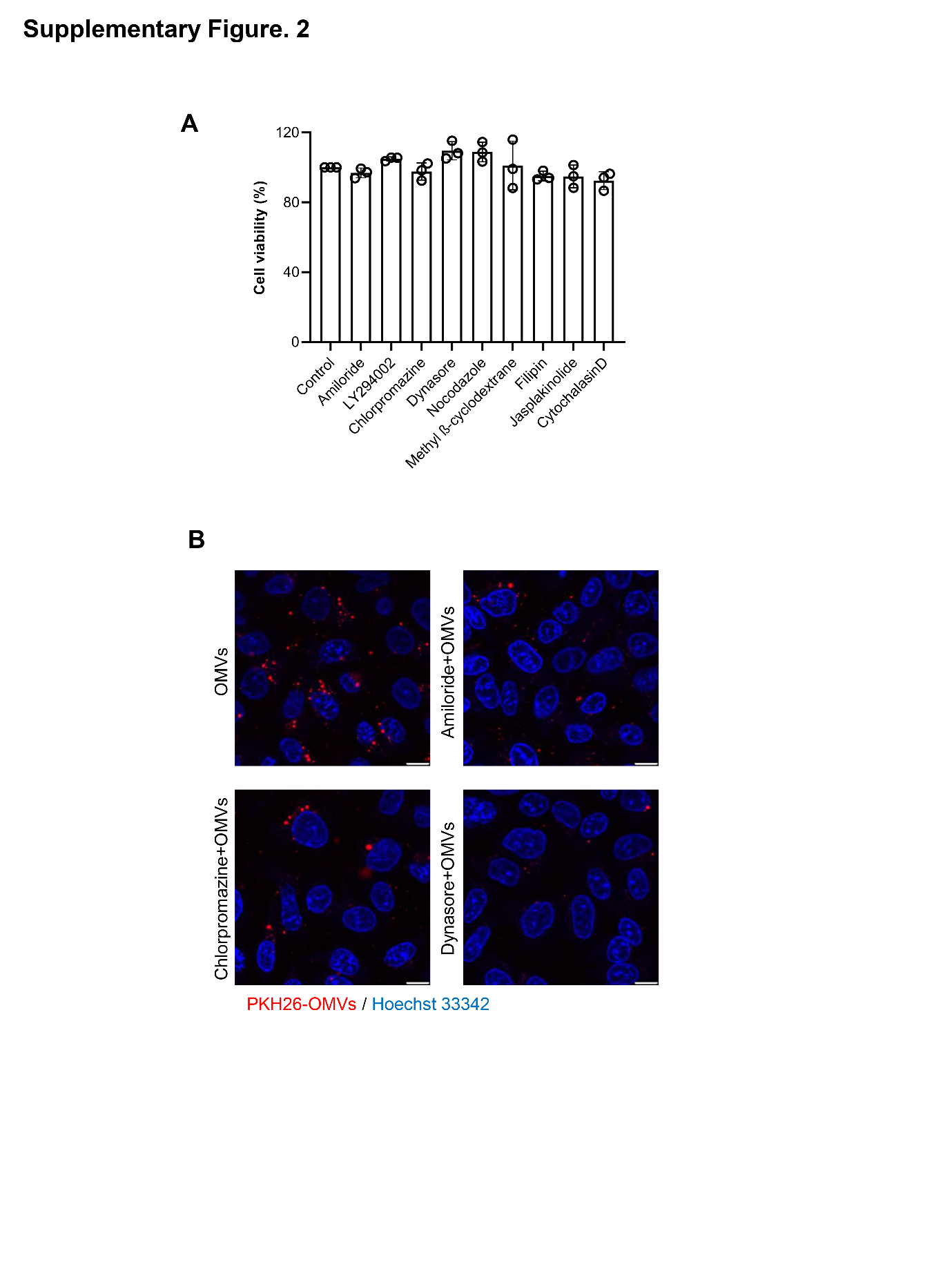


**Figure S3: Internalization of *V. cholerae* OMVs via clathrin-, caveolin- mediated endocytosis, and macropinocytosis.**

**(A)** HCT8 cells were treated with various endocytosis inhibitors: Amiloride (1 mM), LY294002 (20 μM), chlorpromazine (20 μM), Dynasore (50 μM), Nocodazole (20 μM), methyl β-cyclodextrane (5 mM), Filipin (5 μg/mL), Jasplakinolide (500 nM) or CytochalasinD (2 μM) for 4.5 h. Cell viability was accessed using the MTS assay. **(B)** The effect of selected endocytosis inhibitors on the cellular uptake of PKH26-labeled OMVs was further validated through confocal microscopy. Scale bars, 10 μm.


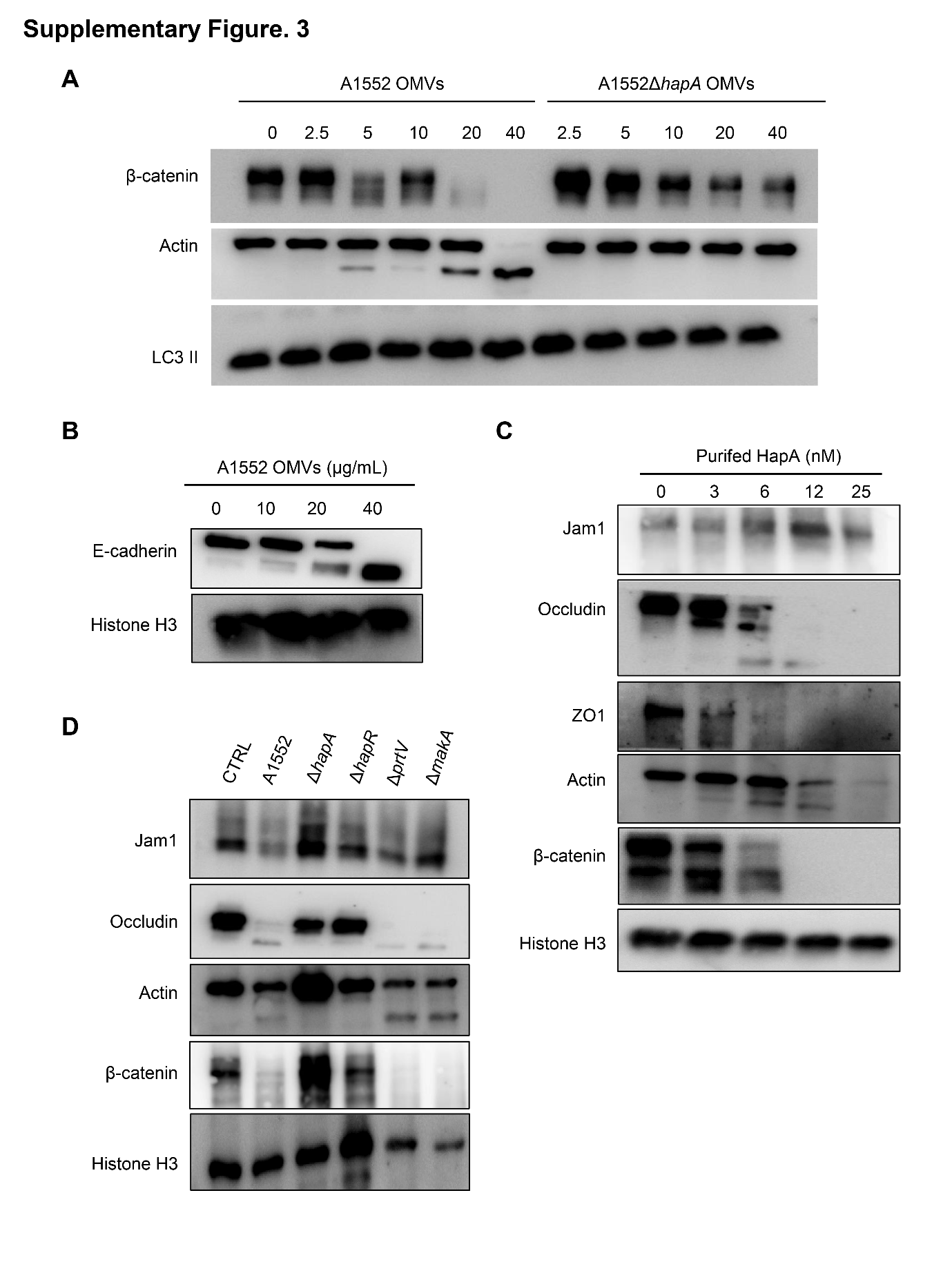


**Figure S4: HapA is primarily responsible for the cleavage of intracellular proteins of the host cells.**

**(A)** Western blot analysis of HCT8 cells exposed to increasing concentrations of OMVs (4 h). *V. cholerae* OMVs induced a concentration-dependent cleavage of tight- and adherens-junction proteins. OMVs were isolated from *V. cholerae* strain, A1552 wild-type (A1552 OMVs) or a strain lacking HapA (Δ*hapA* OMVs). LC3 II was used as a loading control. **(B)** Western blot analysis of HCT8 cells exposed to increasing concentrations of OMVs (4 h) isolated from *V. cholerae* A1552, showing cleavage of the adherens-junction protein E-cadherin. **(C)** Western blot analysis of HCT8 cells exposed to increasing concentrations of purified HapA (4 h). Purified HapA caused concentration-dependent cleavage of tight- and adherens-junction proteins. **(D)** Western blot analysis of HCT8 cells treated with OMVs (20 μg/mL) isolated from wild-type *V. cholerae* A1552 or strains lacking *hapA,* *hapR*, *prtV* or *makA*. OMVs from all strains, except those lacking HapA (Δ*hapA*), caused cleavage of tight- and adherens-junction proteins.


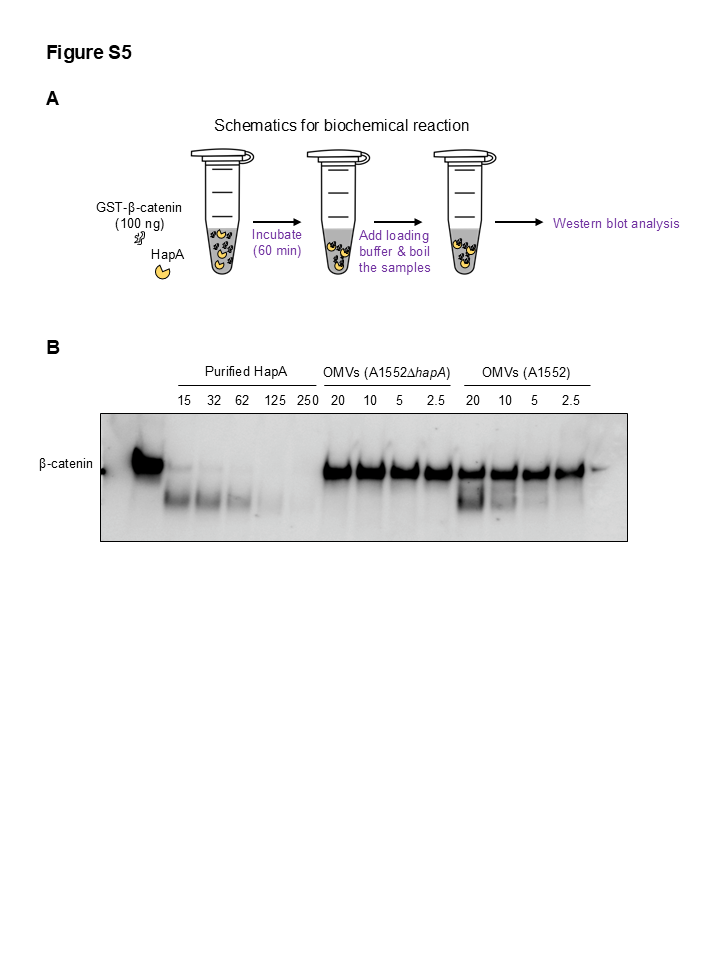


**Figure S5: OMV-associated HapA mediates the cleavage of purified β-catenin.**

**(A)** Schematic overview of the Western blot analysis performed for investigating the cleavage of β-catenin. **(B)** Purified β-catenin is cleaved by soluble and OMVs associated HapA in a concentration dependent manner.


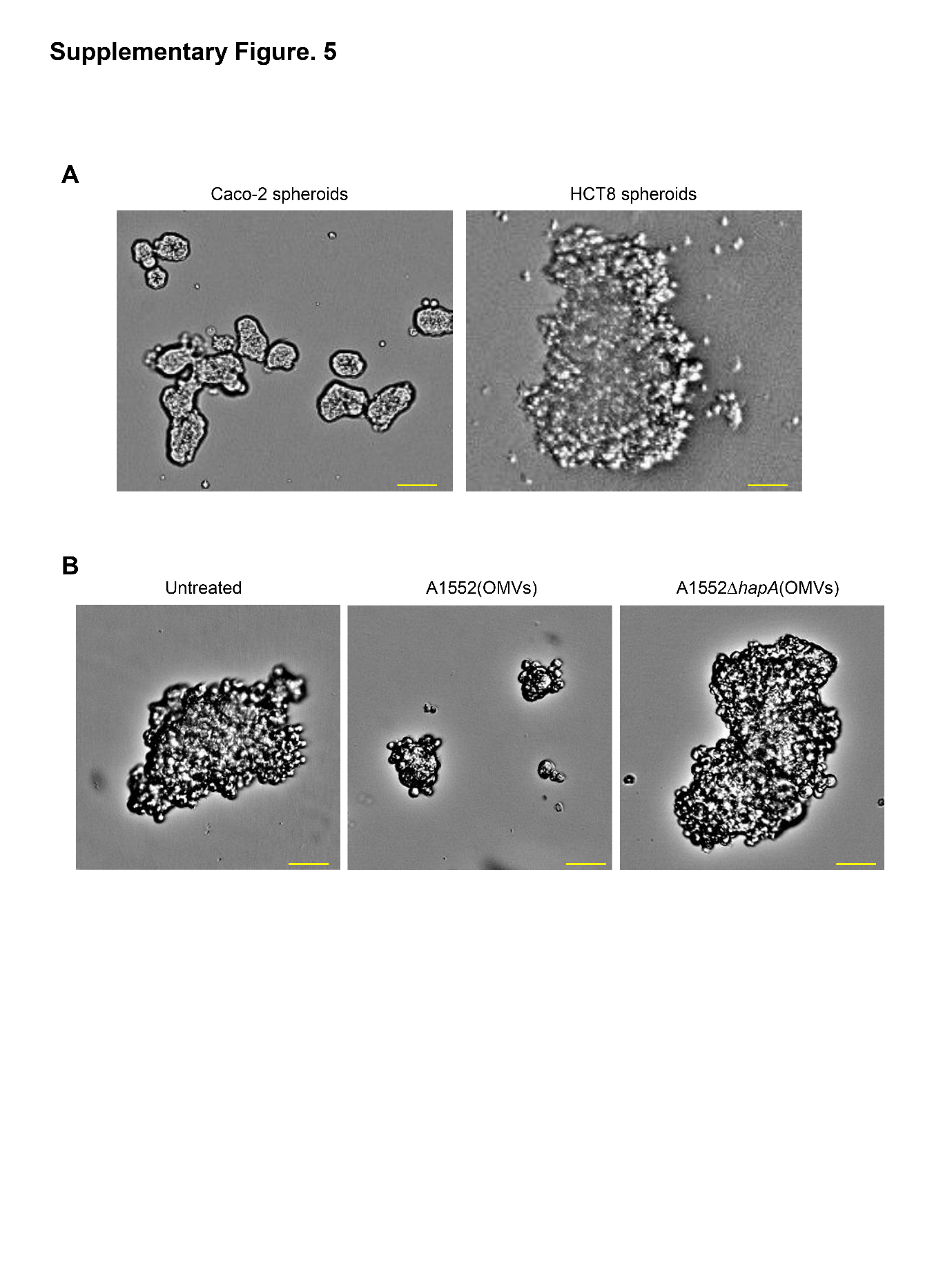


**Figure S6: *V. cholerae* OMV-associated HapA prevents spheroids formation by HCT8 cells.**

**(A)** Comparison of 3D spheroid structures formed by Caco-2 and HCT8 cells. Scale bars, 50 μm **(B)** OMVs isolated from wild-type *A1552* disrupt HCT8 spheroid as compared to the A1552Δ*hapA* OMVs. Scale bars, 50 μm


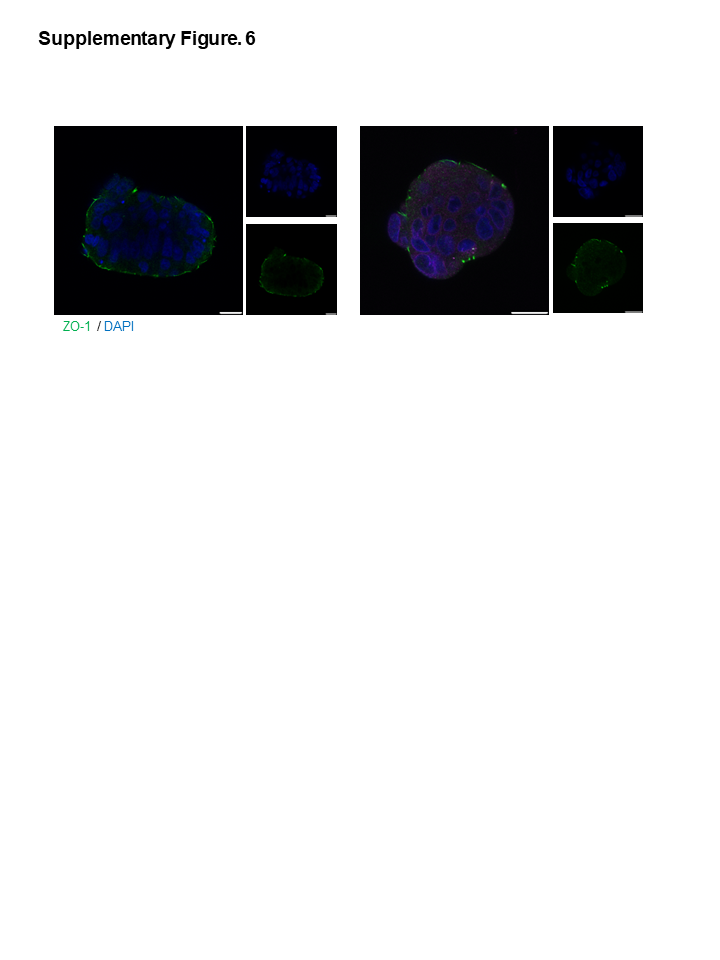


**Figure S7: OMV-associated HapA caused decreased staining of ZO-1 in basal-out intestinal organoids.**

Intestinal organoids were treated with *V. cholerae* OMVs (50 μg/mL) isolated from A1552 wild-type for 18 h, followed by fixation and staining for tight junction proteins, ZO-1 (green), and DAPI (blue). Scale bars, 10 μm.

**Movie Caption**

**Movie S1: *V. cholerae* OMVs co-localizes with caveolin**

This movie was generated from time-lapse confocal fluorescence microscopy images (frame rate, 2 fps; total duration of imaging, 10 min) of HCT8 cells transfected with GFP-Cav1 (green), followed by exposure to PKH26-OMVs (red). Nuclei were counterstained with Hoechst 33342 (blue). Images were acquired 4 h after OMV treatment. Scale bar = 20 μm.
