# Supplementary figures and images for: Hemagglutinin Protease HapA Associated With Vibrio cholerae Outer Membrane Vesicles (OMVs) Disrupts Tight and Adherens Junctions
Source: J Extracell Vesicles. 2025 May 25;14(5):e70092. doi: 10.1002/jev2.70092 (PMC12104216; doi:10.1002/jev2.70092)

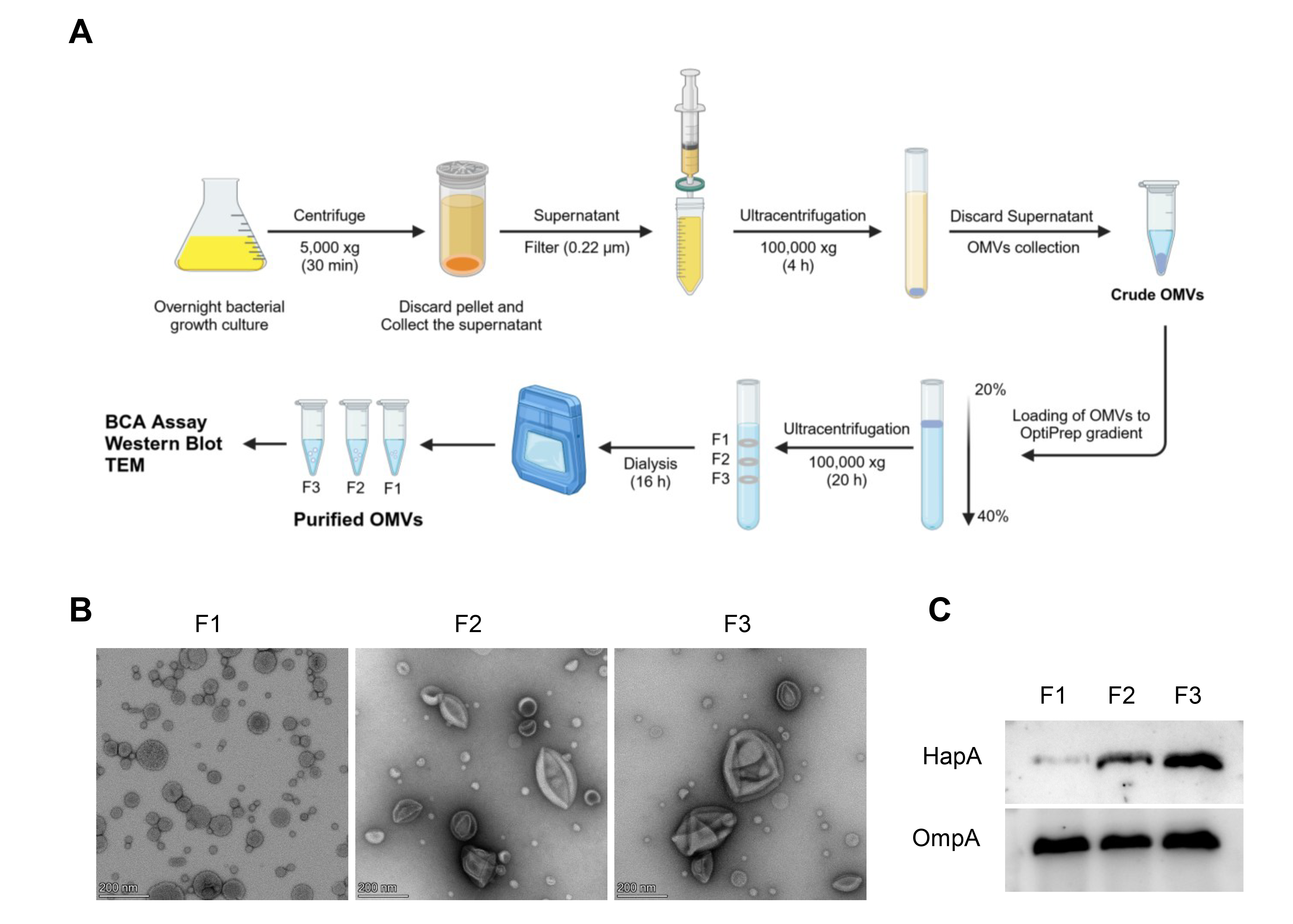

Supplement: Supplementary file 2 — Supporting Information [file JEV2-14-e70092-s006.tif]

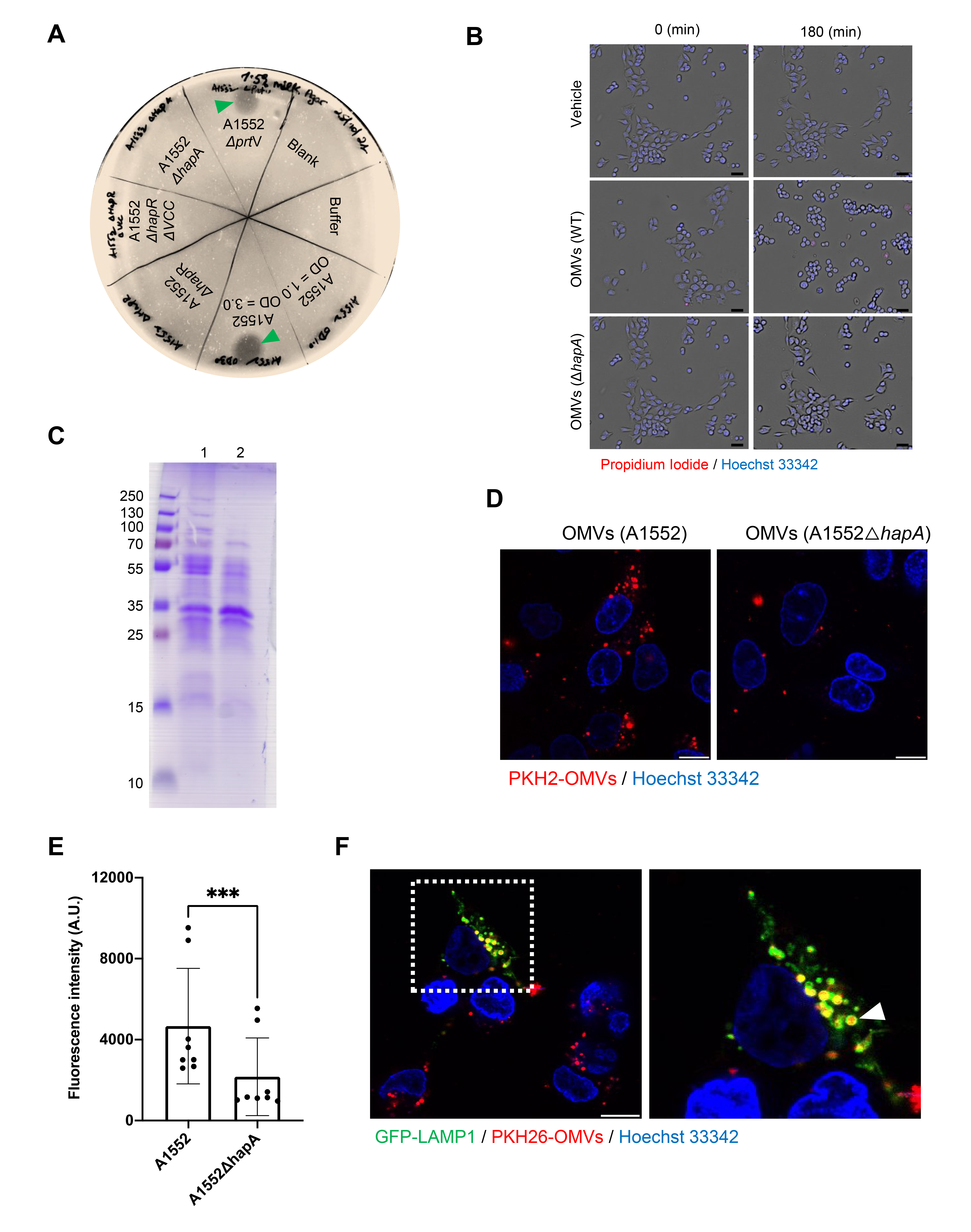

Supplement: Supplementary file 3 — Supporting Information [file JEV2-14-e70092-s008.tif]

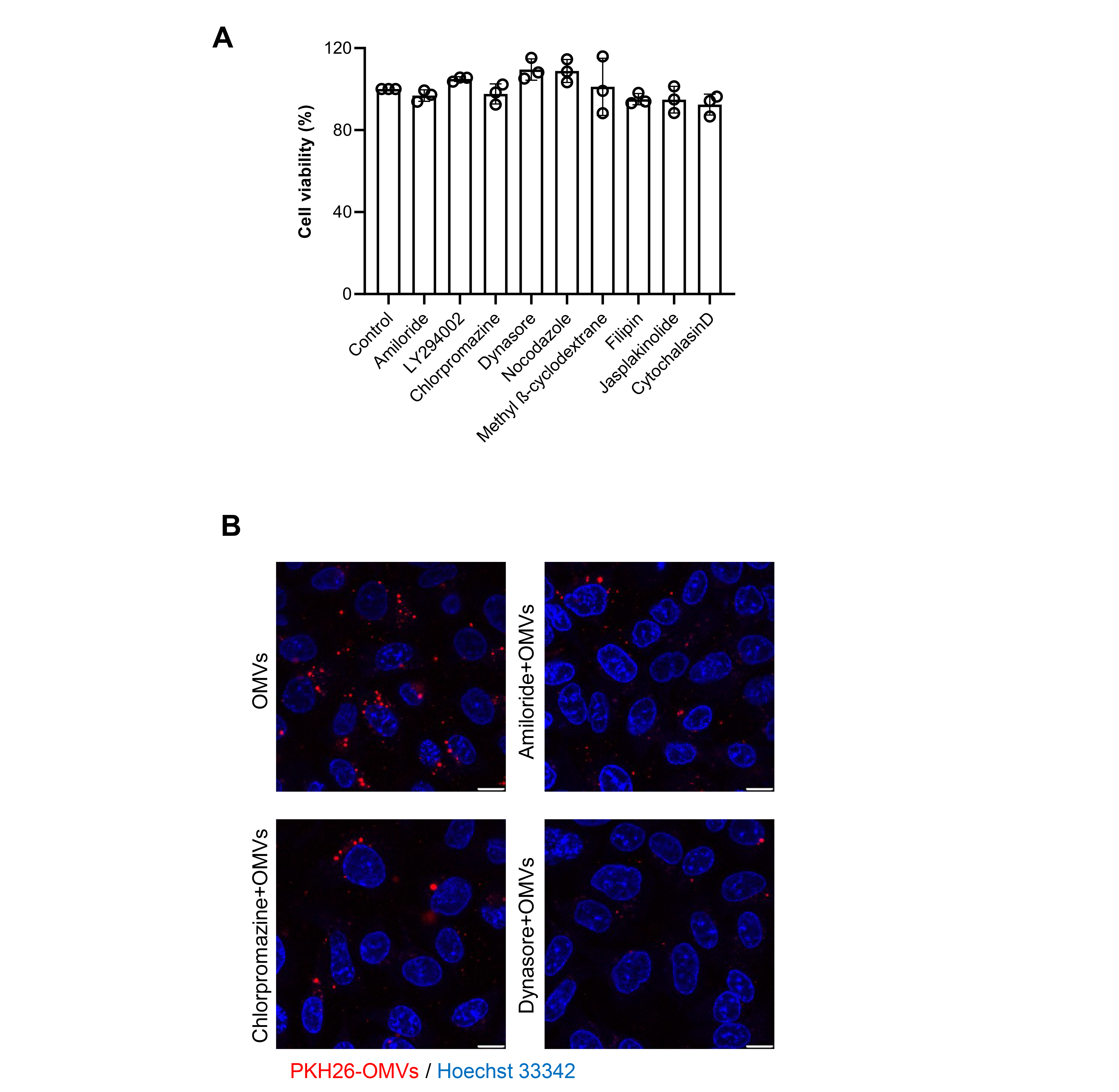

Supplement: Supplementary file 4 — Supporting Information [file JEV2-14-e70092-s003.tif]

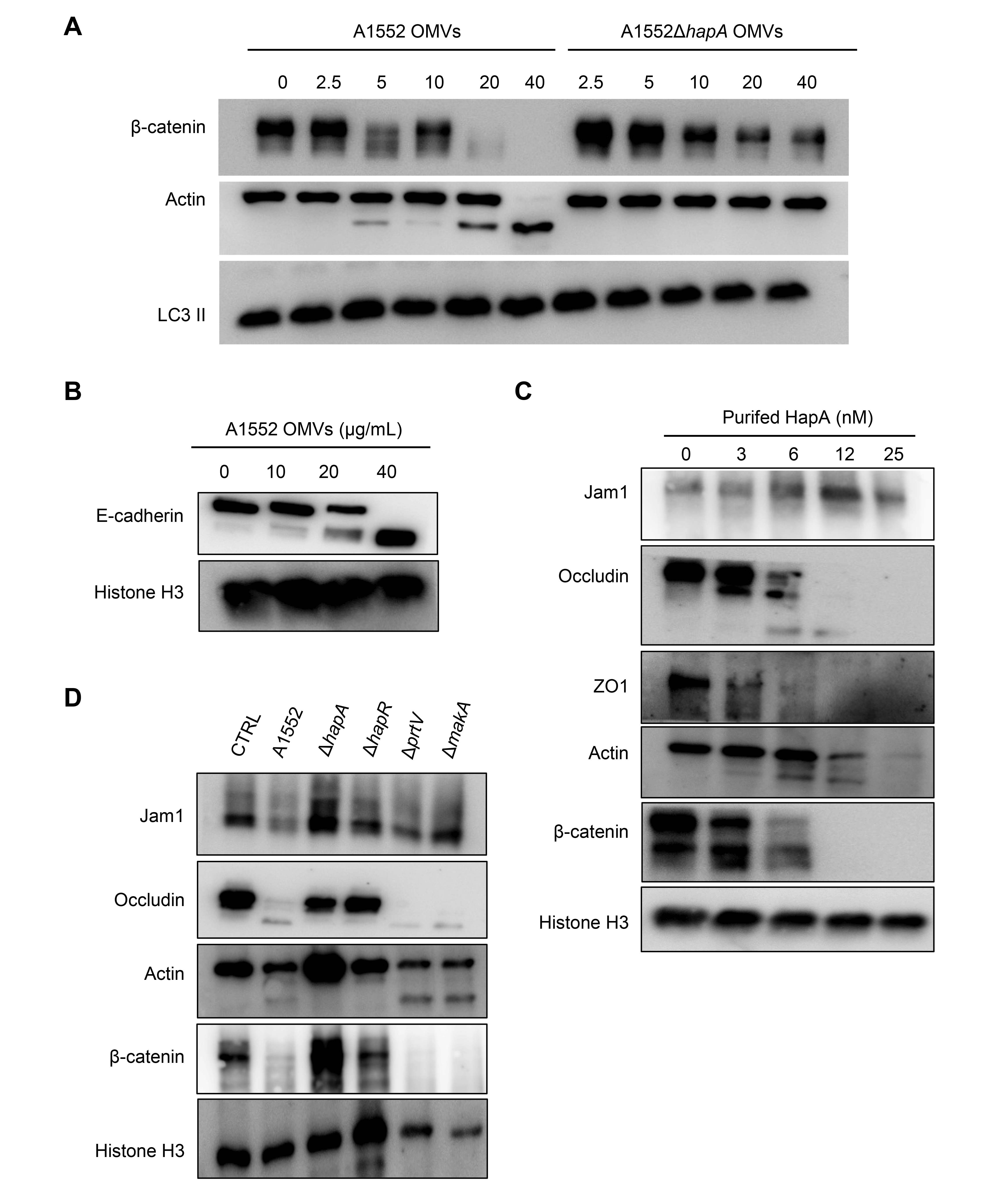

Supplement: Supplementary file 5 — Supporting Information [file JEV2-14-e70092-s009.tif]

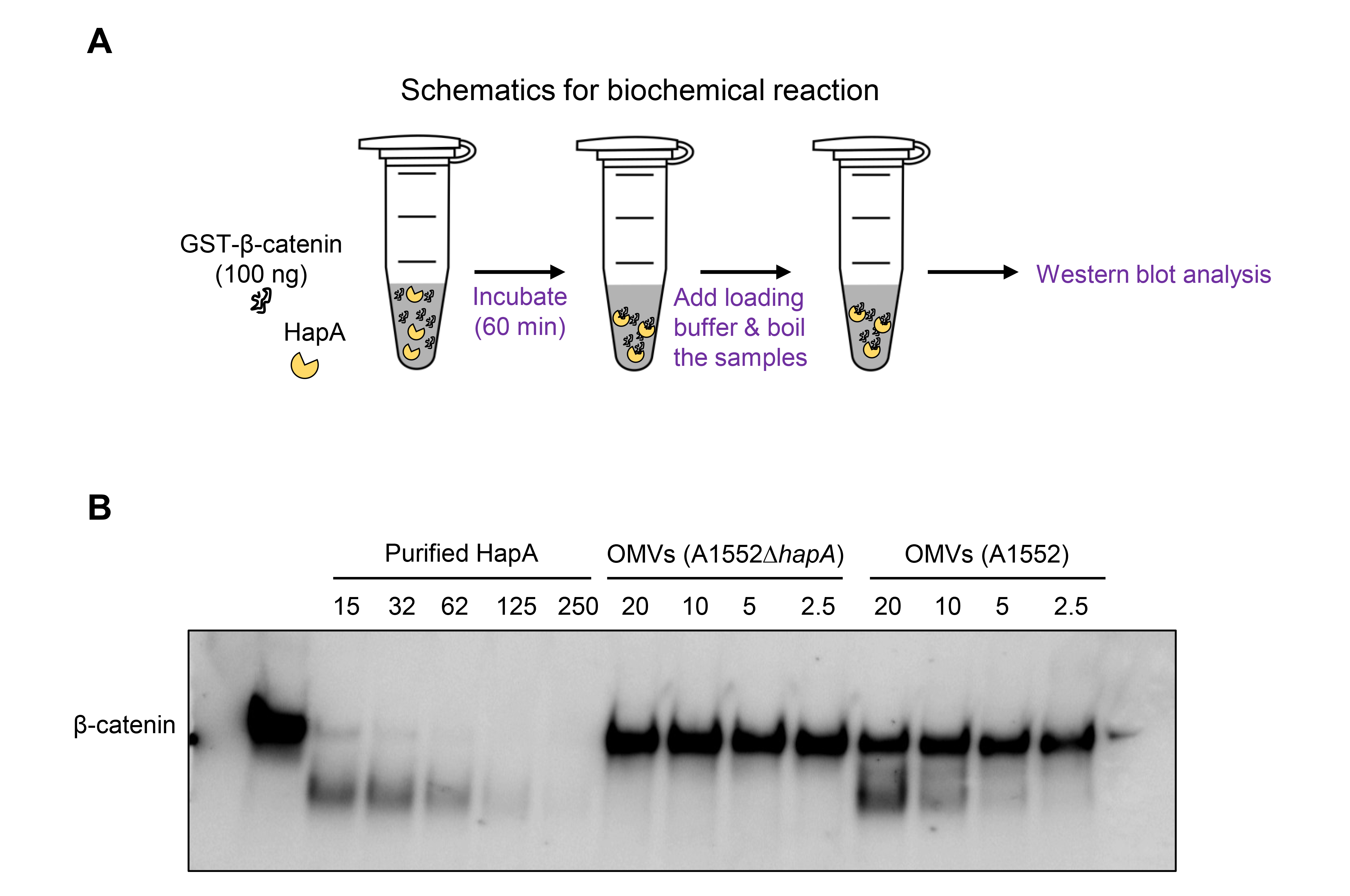

Supplement: Supplementary file 6 — Supporting Information [file JEV2-14-e70092-s005.tif]

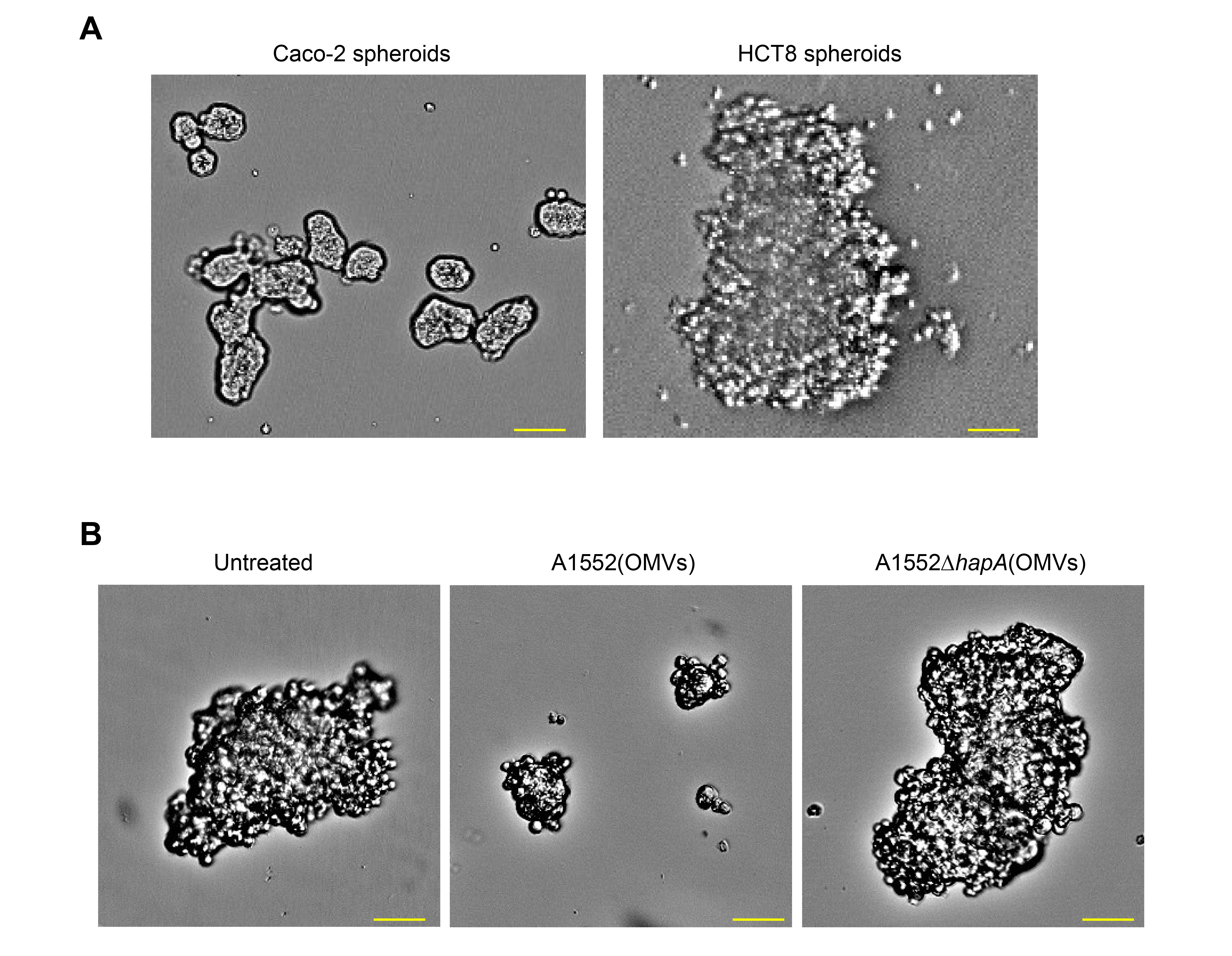

Supplement: Supplementary file 7 — Supporting Information [file JEV2-14-e70092-s004.tif]

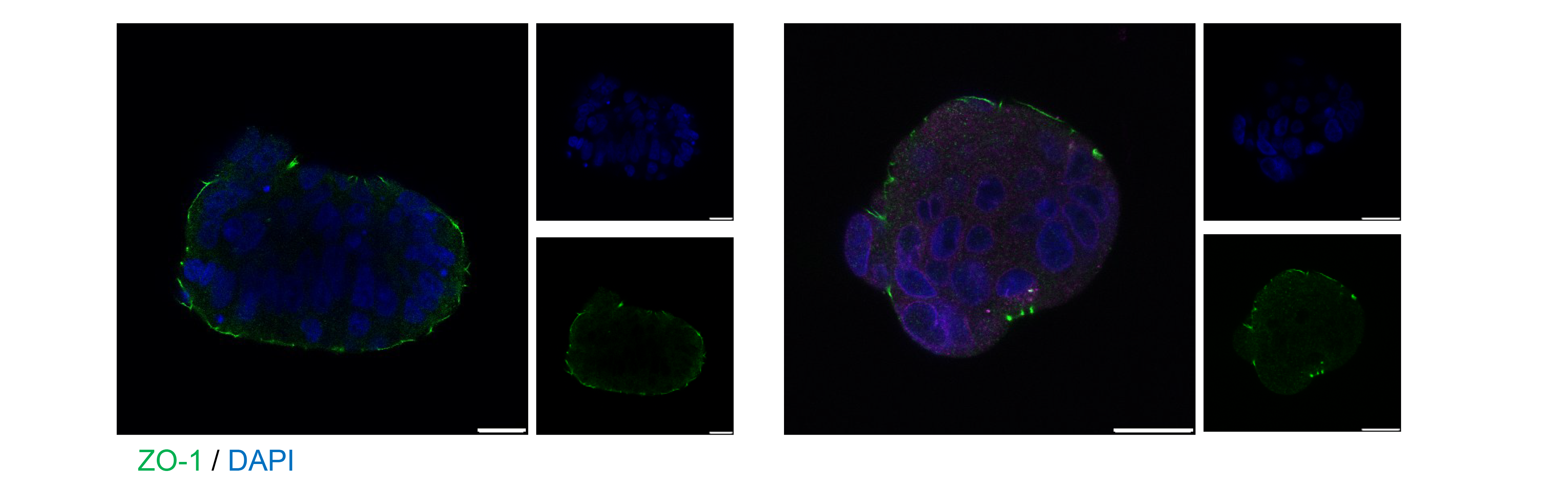

Supplement: Supplementary file 8 — Supporting Information [file JEV2-14-e70092-s007.tif]
